# Comprehensive assessment of fungal peroxygenases for the selective synthesis of 25-hydroxyvitamins D_2_ and D_3_

**DOI:** 10.1186/s13068-026-02792-0

**Published:** 2026-07-10

**Authors:** Alejandro González-Benjumea, Andrés Olmedo, Katharina Ebner, Anton Glieder, Ana Gutiérrez

**Affiliations:** 1 Instituto de Recursos Naturales y Agrobiología de Sevilla (IRNAS), CSIC, Reina Mercedes 10, 41012 Seville, Spain; 2 Bisy GmbH, Hofstätten an der Raab, Austria

**Keywords:** Unspecific peroxygenases, UPOs, *Hypoxylon* sp., *Komagataellaphaffii*, Ergocalciferol, Cholecalciferol, Vitamins

## Abstract

**Background:**

The metabolite 25-hydroxyvitamin D is significantly better for clinical use in several diseases and food supplementations than vitamin D (calciferol) itself. The present study reports an efficient biotechnological method for the synthesis of 25-hydroxyvitamin D_2_ and 25-hydroxyvitamin D_3_, in one step, starting from the readily available and comparatively inexpensive vitamin D_2_ (from fungi and yeast) and vitamin D_3_ (from animals).

**Results:**

A widespread investigation of a series of up to 71 unspecific peroxygenases (UPOs), including UPO samples from 41 fungal species (together with several isozymes and variants) expressed in *Komagataella phaffii* (formerly *Pichia pastoris*), has been performed for the regioselective C25-hydroxylation of vitamin D (D_2_ and D_3_). First experiments revealed that 42 UPOs were able to transform vitamin D_2_ into its 25-hydroxy derivative within a wide range of conversion rates (from 5% to > 99%), with strict regioselectivity in most cases, determined using GC–MS. The 16 best UPOs in terms of conversion and regioselectivity were tested for vitamin D_3_ hydroxylation. With vitamin D_3_, other hydroxylated derivatives were produced by UPOs in addition to the 25-hydroxy derivative, with the only exception of the UPO from *Hypoxylon* sp. (*Hsp*UPO). Finally, the synthesis of both 25-hydroxyvitamin D_2_ and D_3_ was scaled up (up to 20 mM of substrate loading) with *Hsp*UPO, using different cosubstrates, such as H_2_O_2_ or O_2_/reductants, with ascorbic acid, gallic acid, or pyrogallol as reductants. High conversions (68–97% for vitamin D_2_ and 63–75% for vitamin D_3_) and product yields (14–19 mM for vitamin D_2_ and 13–15 mM for D_3_) were attained with all cosubstrates, with pyrogallol/O_2_ and H_2_O_2_ showing the best results with both vitamins.

**Conclusions:**

This approach represents a green and sustainable alternative to the chemical synthesis of these hydroxylated compounds that is tedious, occurs with little regioselectivity, and includes several steps and strong oxidizing agents. Moreover, *K. phaffii* a widely used yeast platform for recombinant protein production ‒ including high-yield secretory expression of industrial enzymes ‒ has been firmly established as a favorable host for recombinant UPO production in recent years.

**Graphical Abstract:**

**Figure:**
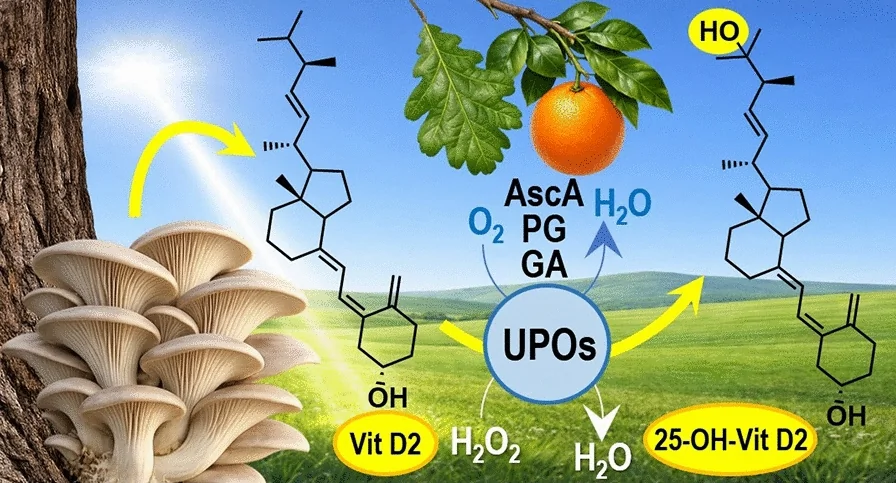

**Supplementary Information:**

The online version contains supplementary material available at https://doi.org/10.1186/s13068-026-02792-0.

## Background

**Fig. 1:**
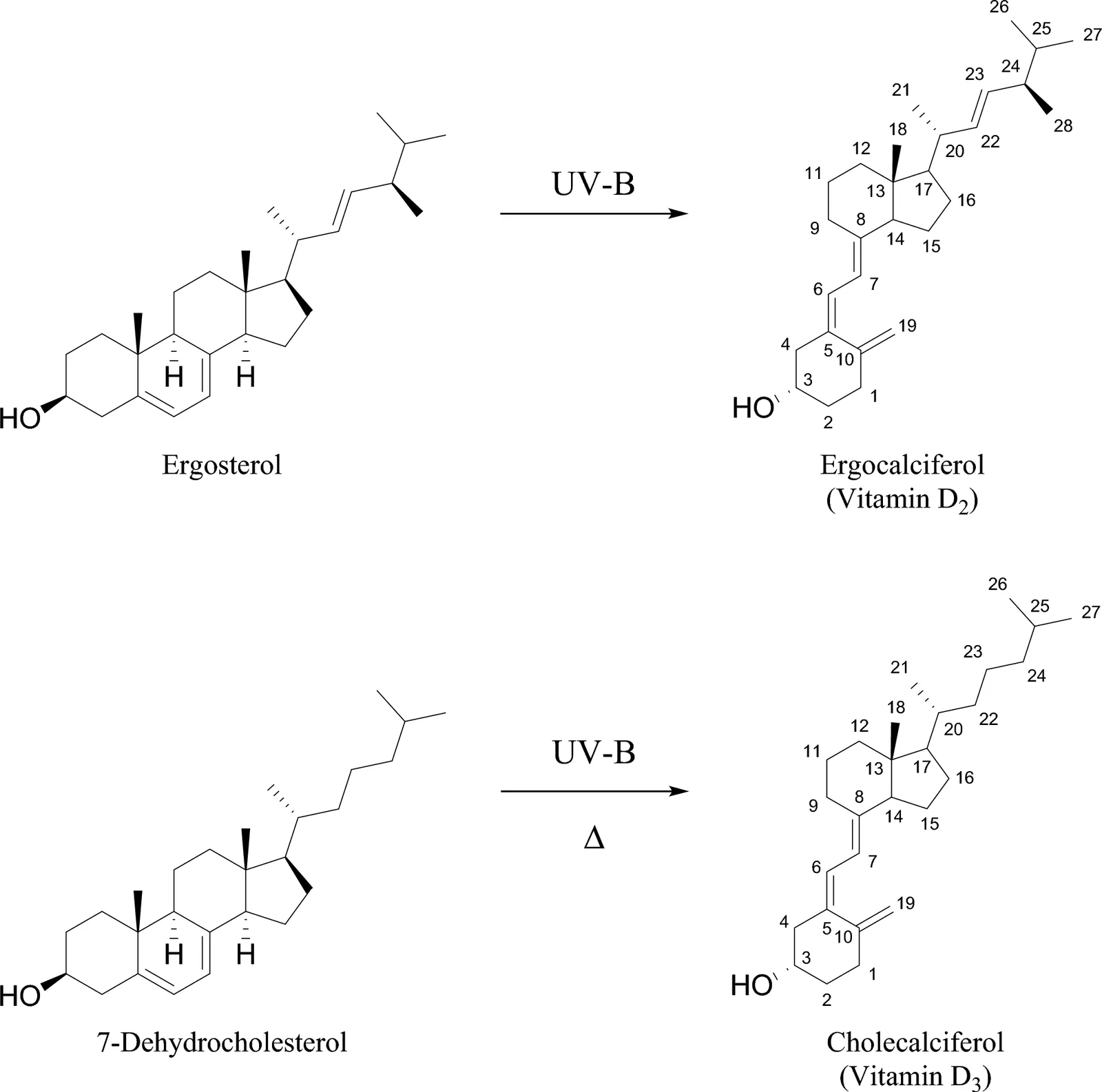
Conversion of ergosterol to vitamin D_2_ (top) and 7-dehydrocholesterol to vitamin D_3_ (bottom)

Vitamin D is a secosteroid compound derived from a broken B-ring steroid skeleton. It plays important roles in health, and its deficiency or insufficiency is accompanied with an increased risk of a plethora of disorders and illnesses such as rickets, osteomalacia, preeclampsia, dental pathologies, cancers, cardiovascular, and autoimmune diseases [1, 2]. In nature it is found in two forms predominantly, vitamin D_2_ (ergocalciferol) and vitamin D_3_ (cholecalciferol), formed from ergosterol (in fungi and yeast) and 7-dehydrocholesterol (in animals), respectively (Fig. 1) [3].

Nevertheless, vitamin D is a prodrug that needs to be transformed in vivo into active forms. These biologically active forms are hydroxylated metabolites at C-25 and C-1 produced by several cytochrome P450s. In vertebrates, vitamin D is metabolized in the liver producing 25-hydroxyvitamin D (25-OH-D), the most abundant metabolite circulating in blood, and then it is subsequently hydroxylated in the kidneys to form 1α,25-dihydroxyvitamin D (1,25-di-OH-D) which is less abundant but much more active [4]. Besides, 25-OH-D is easier absorbed than vitamin D and also its half-life is significantly longer (including 1,25-di-OH-D) [5] becoming it in a convenient candidate for food supplementations [6, 7] and clinical uses in patients with malabsorption of vitamin D or liver diseases [4]. In addition, the use of 25-OH-D as feed additive for poultry [8, 9] and swine [10] nutrition is more and more frequent. Although in most cases the 25-OH-D used for these purposes is produced from vitamin D_3_, the development of new procedures for the synthesis of the D_2_ form could be interesting to obtain the corresponding metabolite from non-animal (vegan) sources since ergosterol is industrially produced from yeasts [11].

Therefore, there is a growing interest in developing sustainable and efficient methods to synthesize 25-OH-D. Several approaches of chemical synthesis from vitamins D_2_ and D_3_ are currently used, which involve tedious and large routes to construct the side-chain and negatively impact the overall yield [12–16]. Biocatalytic synthesis based on whole-cell systems (fungal and bacterial strains) is also extensively studied, in particular for producing 25-OH-D_3_ in a greener and more efficient approach. However, although this strategy improves the conversion of the process, the substrate load is limited and linked to the production of the microbial strain [17–20]. Most recently, enzymatic synthesis using hydroxylases from microbial sources appears as an alternative to chemical and whole-cell syntheses. P450 monooxygenases stand out as one of the most used enzymes but the need of expensive cofactors and a relatively low stability derived from their intracellular nature limit their applicability [21–23]. Remarkably, steroid C25 dehydrogenases, a subfamily of molybdenum enzymes, efficiently produce 25-OH-D_3_ in high yields with the added-value of using K_3_[Fe(CN_6_)] instead of NAD(P)H as electron source. Regrettably, the anaerobic conditions necessary for the catalytic activity of these enzymes and the associated costs hinder their industrial exploitation [24, 25]. Despite the progress achieved in biocatalytic synthesis, to date most vitamins with the exception of vitamins C, B2, and B12 are still manufactured by chemical methods [26].

In this context, unspecific peroxygenases (UPOs, EC. 1.11.2.1), the so-called “dream biocatalysts,” have become promising candidates for the oxyfunctionalization of added-value compounds of industrial interest due to their advantages such as the use of hydrogen peroxide or organic hydroperoxides as the only cosubstrate, a high stability relative to their extracellular nature, and a great tolerance to certain organic solvents [27]. However, up to now only a few studies on enzymatic hydroxylation of vitamin D have been reported, which were mostly focused on the wild-type UPO from *Agrocybe aegerita* (*Aae*UPO, isolated from cultures of the native fungus), or its laboratory evolved variant PaDa-I [28–32] since the widespread use of UPOs in the field of biocatalysis was hindered by their arduous heterologous production, considerably limiting the panel of enzymes available for investigation and enzyme engineering. Fortunately, very recently, the heterologous expression of UPOs has been successfully established and improved in different recombinant hosts improving their production and enabling the access to engineered variants [33–37]. Particularly, *Komagataella phaffii* (formerly *Pichia pastoris*), a widely used yeast platform for recombinant protein production ‒ including high-yield secretory expression of industrial enzymes ‒ has been firmly established as a favorable host for recombinant UPO (rUPO) production in recent years. So far, more than 60 different UPO sequences (short- and long-type enzymes) have been recombinantly produced using *K. phaffii* [33, 37–40] with secretory protein amounts up to 742 mg/L published for short-type UPOs [41].

Taking advantage of these advances in enzyme expression, in the present work we evaluate for the first time a panel of up to 71 UPO samples produced in *K. phaffii*, for the selective hydroxylation of vitamin D_2_ and vitamin D_3_. Next, the hydroxylation of both vitamin D forms is upscaled with a representative UPO using two different procedures. Several reaction parameters such as cosolvent, solubilizing additive, enzyme dose, and reaction time were optimized to achieve high conversions and regioselectivity. All the UPO reactions with vitamins D_2_ and D_3_ were analyzed by GC–MS.

## Materials and methods

### Chemicals

Vitamins D_2_ and D_3_ as well as their C-25 hydroxylated metabolites (25-OH-D_2_ and 25-OH-D_3_) were acquired from Merck. Their corresponding C-1 hydroxylated metabolites (1-OH-D_2_ and 1-OH-D_3_) were acquired from TCI and the calcitriol derivatives (1,25-di-OH-D_2_ and 1,25-di-OH-D_3_) were purchased from Alfa. The partially methyl and 2-hydroxypropyl-β-cyclodextrin (MCD and HPCD) were purchased from Merck and the sulfobutylether-β-cyclodextrin (SBECD) from Targetmol. Hydrogen peroxide solution (35%) was acquired from Panreac. The reducing agents, namely, ascorbic acid (AscA), gallic acid (GA), and pyrogallol (PG), were obtained from Merck. The derivatizing agent *N*,*O*-bis(trimethylsilyl)trifluoroacetamide (BSTFA) was also purchased from Merck. The organic solvents (GC or HPLC grade) were obtained from commercial suppliers and used without further purification.

### Enzymes

Expression of enzyme sequences was done as described previously, and information on all tested UPOs is given in Table 1. Bioreactor scale UPO production was adapted from Rotilio et al*.* [37, 42] employing a fed-batch bioprocess and lyophilization of the resulting enzyme-containing supernatant. The bioprocess was downscaled employing a small-scale parallel bioreactor system (DASBox, Eppendorf) with a working volume of 250 mL and using the batch and fed-batch medium described by Maurer et al*.* [43]. Enzyme concentrations were estimated by UV–Vis spectroscopy from the reduced-UPO adducts with CO [44].

**Table 1:** UPO samples tested in the present study including wild-type UPOs and isozymes (roman numerals) and variants

Organism of origin	Abbreviation	Organism of origin	Abbreviation
*Aspergillus aculeatus*	*Aac*UPO	*Hypoxylon pulicicidum*	*Hpu*UPO
*Agrocybe aegerita*	*Aae*UPO	*Hypoxylon pulicicidum*	*Hpu*UPO-I
*Aspergillus bombycis*	*Abo*UPO	*Hypoxylon* sp.	*Hsp*UPO
*Aspergillus brasiliensis*	*Abr*UPO-I	*Hypoxylon* sp.	*Hsp*UPO_11H12
*Aspergillus brasiliensis*	*Abr*UPO-II	*Hypoxylon* sp.	*Hsp*UPO_18G3
*Aspergillus ellipticus*	*Ael*UPO	*Hypoxylon* sp.	*Hsp*UPO_20H11
*Armillaria gallica*	*Aga*UPO	*Hypoxylon* sp.	*Hsp*UPO_20H3
*Aspergillus luchuensis*	*Alu*UPO	*Hypoxylon* sp.	*Hsp*UPO_23E12
*Aspergillus niger*	*Ani*UPO-II	*Hypoxylon* sp.	*Hsp*UPO_8G3
*Aspergillus novoparasiticus*	*Ano*UPO	*Hypoxylon* sp.	*Hsp*UPO_V1
*Aspergillus oryzae*	*Aor*UPO	*Hypoxylon* sp.	*Hsp*UPO_V2
*Aspergillus pseudotamarii*	*Aps*UPO	*Hypoxylon* sp.	*Hsp*UPO_V3
*Armillaria solidipes*	*Aso*UPO	*Hypoxylon* sp.	*Hsp*UPO_V5
*Aspergillus tubingensis*	*Atu*UPO	*Hypoxylon* sp.	*Hsp*UPO-II
*Candolleomyces aberdarensis*	*Cab*UPO-III	*Hortaea werneckii*	*Hwe*UPO
*Candolleomyces aberdarensis*	*Cab*UPO-IV	*Laccaria amethystina*	*Lam*UPO
*Candolleomyces aberdarensis*	*Cab*UPO-V	*Leucoagaricus* sp.	*Lsp*UPO-II
*Candolleomyces aberdarensis*	*Cab*UPO-VI	*Marasmius fiardii*	*Mfi*UPO
*Candolleomyces aberdarensis*	*Cab*UPO-VII	*Marasmius fiardii*	*Mfi*UPO-I
*Coprinopsis marcescibilis*	*Cma*UPO-I	*Marasmius nigripes*	*Mni*UPO
*Coprinopsis marcescibilis*	*Cma*UPO-II	*Marasmius oreades*	*Mor*UPO
*Coprinellus micaceus*	*Cmi*UPO	*Marasmius oreades*	*Mor*UPO_V1
*Dendrothele bispora*	*Dbi*UPO	*Marasmius oreades*	*Mor*UPO_V4
*Dendrothele bispora*	*Dbi*UPO-IV	*Marasmius oreades*	*Mor*UPO_V5
*Dendrothele bispora*	*Dbi*UPO-V	*Mycena venus*	*Mve*UPO
*Dendrothele bispora*	*Dbi*UPO-VI	*Podospora anserina*	*Pan*UPO
*Dendrothele bispora*	*Dbi*UPO-VII	*Pseudogymnoascus* sp.	*Psp*UPO-II
*Daldinia* sp.	*Dsp*UPO-I	*Rosellinia necatrix*	*Rne*UPO
*Daldinia* sp.	*Dsp*UPO-II	*Stachybotrys chartarum*	*Sch*UPO
*Eutypa lata*	*Ela*UPO	*Sphaerobolus stellatus*	*Sst*UPO-II
*Gymnopilus dilepis*	*Gdi*UPO-I	*Sphaerobolus stellatus*	*Sst*UPO-III
*Gymnopilus dilepis*	*Gdi*UPO-II	*Sistotremastrum suecicum*	*Ssu*UPO
*Gymnopus luxurians*	*Glu*UPO	*Verticillium nonalfalfae*	*Vno*UPO
*Galerina marginata*	*Gma*UPO-I	*Viridothelium virens*	*Vvi*UPO
*Galerina marginata*	*Gma*UPO-II	*Xylaria multiplex*	*Xmu*UPO
*Hypoxylon fragiforme*	*Hfr*UPO		

The majority of the wild-type UPOs tested in this work have already been described in literature and are publicly available under their respective scientific names [33, 37, 40–42, 45, 46]. In addition, for wild-type UPOs and mutant enzymes used for the first time in this study and considered relevant to the main findings because they showed substrate conversion, details including gene accession numbers and, where applicable, the positions of the mutations introduced in the corresponding enzyme variants are provided in supplementary material (Table S1).

### Enzymatic reactions

#### *Screening of 71 UPOs for vitamin D hydroxylation*

The reactions were conducted with 0.05 mM of vitamin D, 4 µM of UPO and 2.5 mM of H_2_O_2_ in 1 mL of phosphate buffer (50 mM, pH 7) containing acetone as cosolvent (40% v/v). For this purpose, stock solutions of vitamins D (5 mM) were freshly prepared in acetone. An aliquot of the corresponding vitamin D solution (10 µl, 50 nmol) was added to the buffer/acetone mixture followed by the UPO solutions (4 nmol). The resulting solutions were stirred at 30 ºC and the H_2_O_2_ solution (25 µl, 100 mM) was gently added with a syringe pump over 50 min, and then for a further 10 min. The reactions were extracted with methyl *tert*-butyl ether (MTBE) and derivatized with BSTFA/pyridine for GC–MS analyses. Additionally, reactions with *Hsp*UPO using calcidiols as substrates to investigate the formation of calcitriols were conducted under the same conditions. Control experiments were performed without enzyme. The reactions were mostly single-point determinations, although some replicates were performed in some selected cases, and a deviation lower than 10% was produced in all cases.

#### *Scale-up of vitamin D hydroxylation with *Hsp*UPO*

For the scale-up reactions two different cosubstrates were evaluated, namely, H_2_O_2_ and O_2_/reductant. Three reductants, namely, ascorbic acid, gallic acid, and pyrogallol, were tested. Stock solutions of vitamins D (100 mM) and cyclodextrins (50% p/v) were freshly prepared in acetone and sodium phosphate buffer (50 mM, pH 7), respectively. In both procedures, the appropriate aliquot of the corresponding vitamin D solution was dried under nitrogen stream and redissolved in sodium phosphate buffer (50 mM, pH 7) containing the cyclodextrins (MCD, HPCD or SBECD) and the cosolvent (Tables S3, 2 and 3). The UPO solution was added and the mixture was stirred and heated at 30 ºC for 10 min to ensure the solubilization of the substrate. When H_2_O_2_ was used, the UPO solution was added in a split dose and the second one was added after 2 h. Besides, the H_2_O_2_ solution was added using a syringe pump over 4 h and then, the reaction mixtures were stirred under the same conditions for a further 20 h. When O_2_/reductant was used, the UPO solution was added in a single dose. In all cases, the total volume was 250 µL. The reactions were extracted with MTBE and derivatized with BSTFA/pyridine for GC–MS analyses. Control experiments were performed without enzyme. Reactions at substrate concentrations higher than 10 mM were carried out in duplicates and a deviation lower than 5% was observed in all cases.

### GC–MS analyses

GC–MS analyses were performed using a Shimadzu GC–MS QP2010 Ultra equipment, with a DB-5HT (15 m × 0.25 mm i.d. and 0.1 μm film thickness) capillary column. Helium was used as carrier gas at a flow of 0.70 mL min^−1^. The injector was set at 300 °C, and the oven was programmed starting from 150 °C (1 min) to 200 °C at a rate of 10 °C min^−1^ and then to 290 ºC (7 min) at a rate of 20 °C min^−1^. The transfer line was set at 300 °C and the ion source at 280 ºC. Compounds were identified as a pair of pyroisomers by mass spectrometry and/or with authentic standards, and the quantification was carried out from total-ion peak summed areas.

## Results and discussion

### Assessment of UPOs in the hydroxylation of vitamins D_2_ and D_3_

**Fig. 2:**
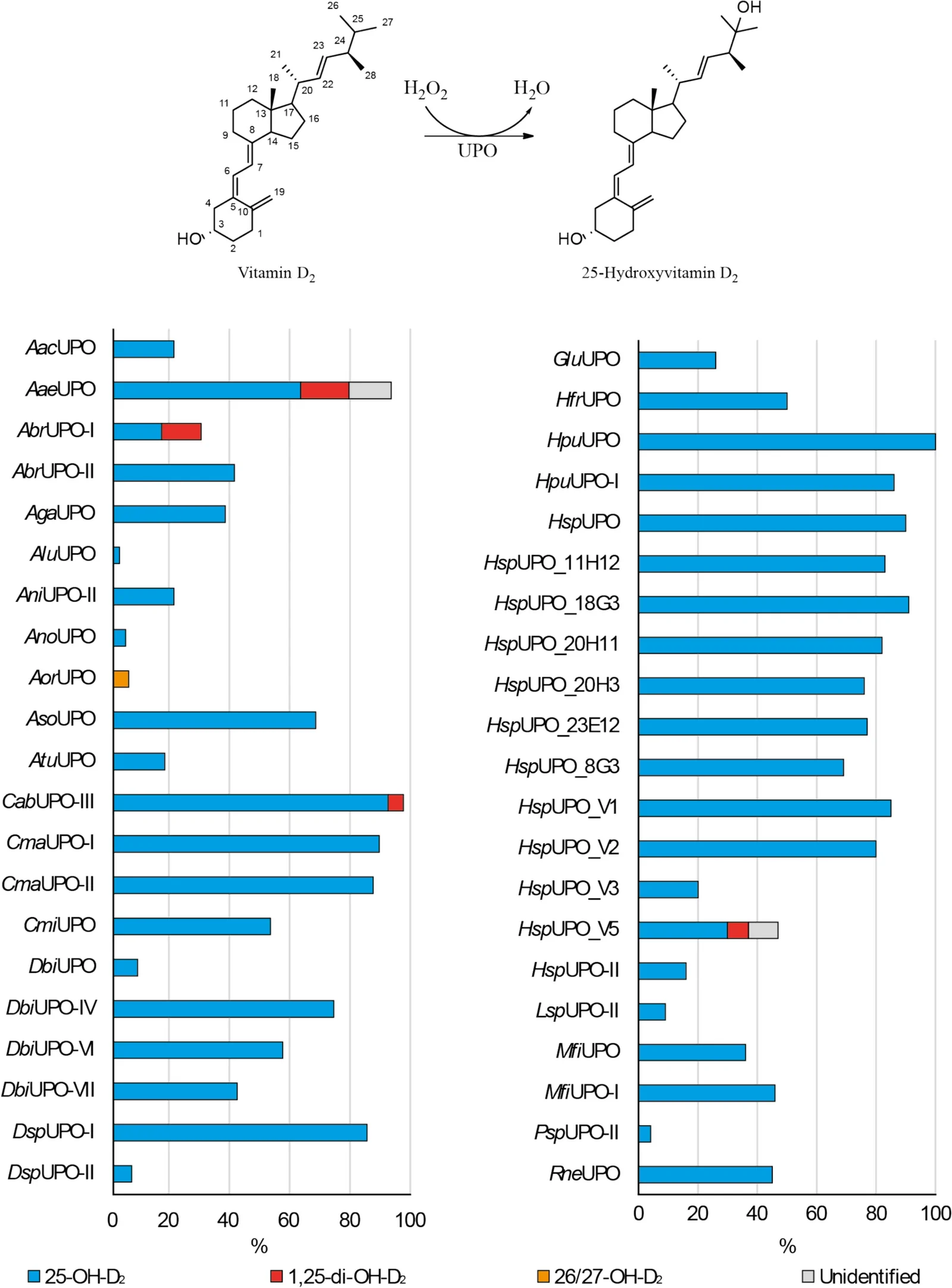
Vitamin D_2_ (0.05 mM) conversion (%) by several UPOs (4 µM) and 2.5 mM H_2_O_2_ within 1 h, and relative percentage of products

Up to 71 UPO samples, which included enzymes from 41 different fungal species, and several isozymes and variants from some of these species, produced with *K. phaffii* (Table 1), were tested with vitamin D_2_ (D_2_) and the results were evaluated using GC–MS (Fig. S1, Table S2). First, to assess the high number of UPO samples we assayed short-term reactions (1 h) with low substrate loading (0.05 mM), 4 µM UPO and 2.5 mM of H_2_O_2_. The GC–MS results (Fig. 2) revealed that 42 UPOs out of 71 transformed D_2_ into its hydroxylated derivative at C25 position with strict regioselectivity in most cases.

Five UPOs, namely, *Aae*UPO, *Cab*UPO-III, *Hpu*UPO, *Hsp*UPO, and *Hsp*UPO_18G3, achieved a very high substrate conversion (≥ 90%) with strict regioselectivity toward C25 position, with the exception of *Aae*UPO and *Cab*UPO-III. Seven UPOs, namely, *Cma*UPO-I, *Cma*UPO-II, *Dsp*UPO-I, *Hpu*UPO-I, *Hsp*UPO_V1, *Hsp*UPO_V2, and *Hsp*UPO_20H11, reached high conversions (82%–88%), also with strict regioselectivity toward C25. Five UPOs, namely, *Aso*UPO, *Dbi*UPO-IV, *Hsp*UPO_8G3, *Hsp*UPO_23E12, and *Hsp*UPO_20H3, attained moderate to good conversions (69%–77%) and strict regioselectivity. The rest of UPOs exhibited low or very low conversions of D_2_.

**Fig. 3:**
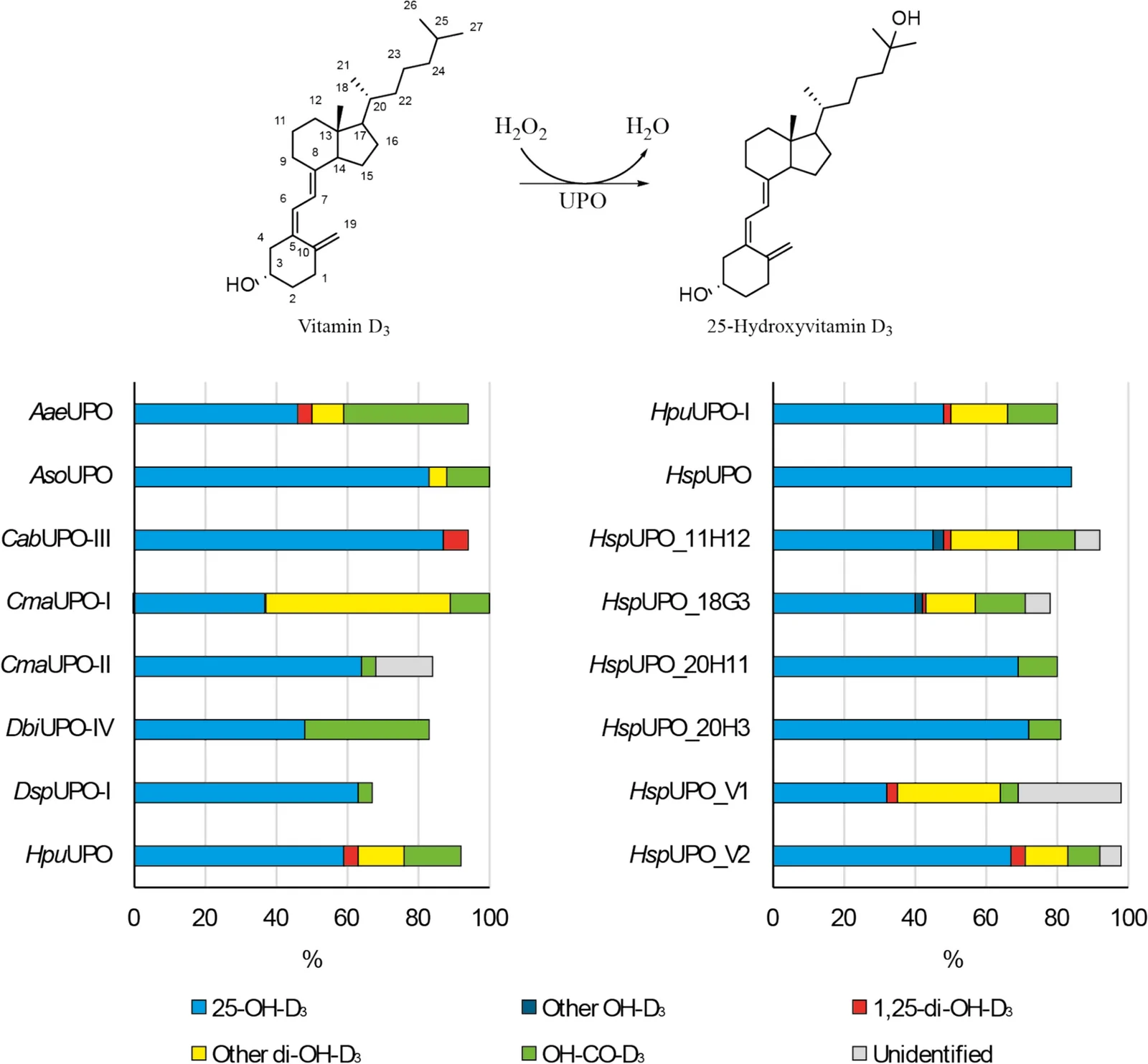
Vitamin D_3_ (0.05 mM) conversion (%) by several UPOs (4 µM) and 2.5 mM H_2_O_2_ within 1 h, and relative percentage of products

We first tested all UPO samples with D_2_ assuming that the enzymes showing good conversions with this vitamin will achieve at least similar performance with D_3_, due to the less bulky side-chain structure in the latter (absence of methyl group C28), as previously observed [28, 29] using r*Cci*UPO and *Aae*UPO. Thereafter, in view of the results, we selected 16 UPO samples, which showed ≥ 80% vitamin D_2_ conversion or > 70% conversion together with very high regioselectivity, and tested them with vitamin D_3_ (D_3_). The reactions were analyzed by GC–MS (Fig. S2, Table S3). Overall, these UPOs also transformed D_3_ with high conversion in most cases (> 80%) and yielded different amounts of the desired product, 25-OH-D_3_ (Fig. 3).

Unlike with D_2_, these enzymes demonstrated higher promiscuity with D_3_, since they promoted its hydroxylation at different positions, and delivered di- and tri-hydroxylated compounds and other products, presumably derived from overoxidation processes. This different behavior may be related to the presence in D_2_ of a methyl group in C-24 and a double bond in C-22–C-23 that causes a steric hindrance that limits the formation of possible conformers.

**Fig. 4:**
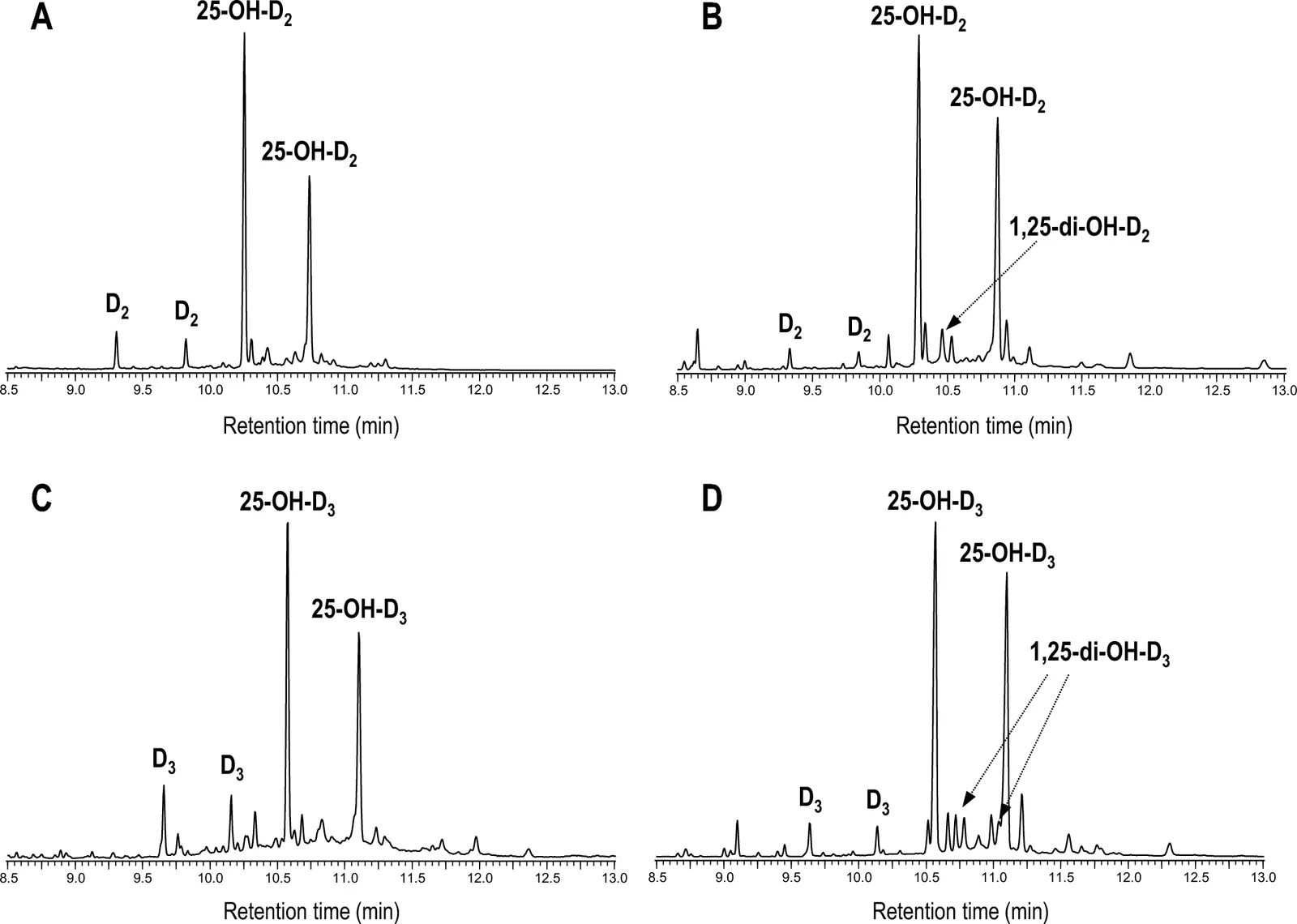
GC–MS analyses of UPO reactions (as TMS derivatives) with vitamin D_2_ (top) and vitamin D_3_ (bottom) catalyzed by *Hsp*UPO (left, **A** and **C**) and *Cab*UPO-III (right, **B** and **D**) producing 25-hydroxyvitamins D (25-OH-D_2_ and D_3_) as the main product. The isopyro (left) and pyro (right) isomers from secosteroid thermal rearrangement are obtained

Although in several reactions, the position of the additional hydroxy groups is unclear, calcitriol (C-1,25 hydroxylation) has been identified among the regioisomers in half of the reactions. Nevertheless, *Hsp*UPO and *Cab*UPO-III were the two best UPOs, in terms of regioselectivity toward 25-OH-D_3,_ which was completely strict in the case of *Hsp*UPO, and the two UPOs that showed more similar performance with D_3_ and D_2_ (Fig. 4). Despite the existence of many more oxygenated derivatives of D_3_, only a few are reported to have a clear biological functionality, in addition to the well-known 1,25-di-OH-D_3_ derivative. Therefore, 21,25-di-OH-D_3_ has been reported to have a preferential activity in mobilizing calcium from the bone, while 25,26-di-OH-D_3_ appears to have its greatest effect in stimulating intestinal calcium transport [47]. On the other hand, 24,25-di-OH-D_3_ is commonly considered as biomarker of vitamin D degradation. Additionally, some evidences of its role in bone formation and lower bone fracture risk are reported [48]. According to this, the unidentified byproducts obtained in our reactions might have similar or resembling activities, although a complete characterization of their structures together with the biological evaluation would be required.

**Fig. 5:**
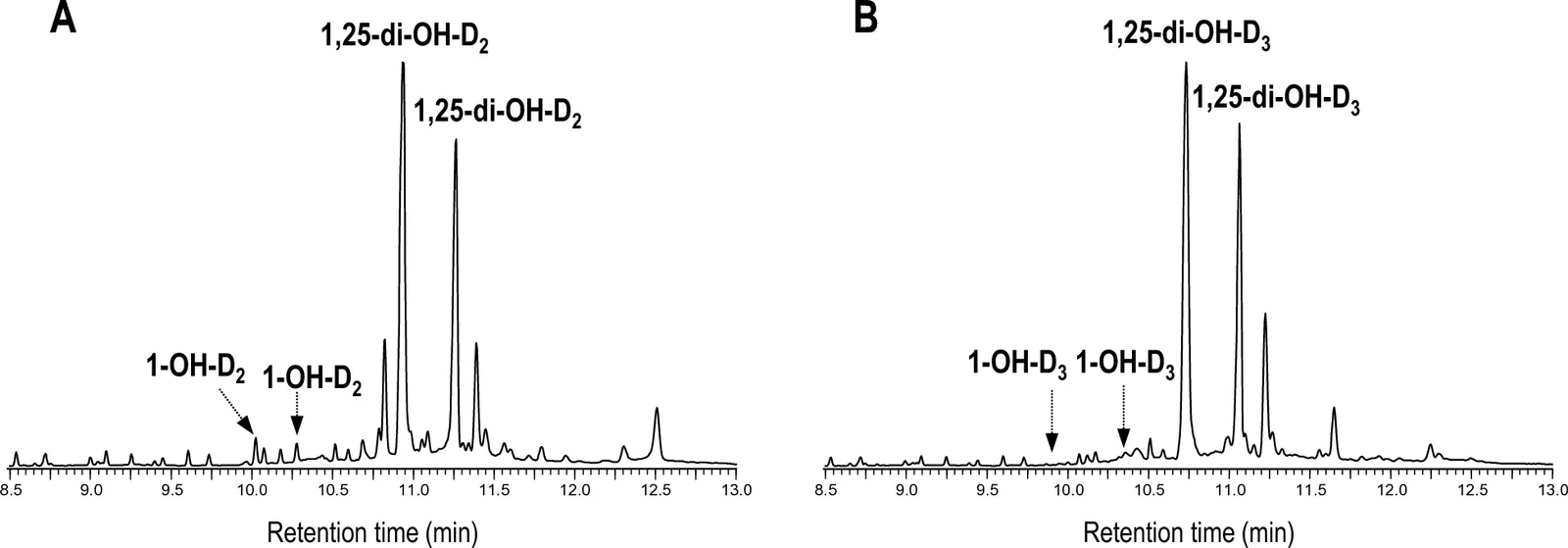
GC–MS analyses of UPO reactions (as TMS derivatives) with 1-hydroxyvitamin D_2_ (*left*) and 1-hydroxyvitamin D_3_ (*right*) and *Hsp*UPO that yielded the corresponding calcitriols. The isopyro (left) and pyro (right) isomers from secosteroid thermal rearrangement are obtained

Although calcitriol (1,25-di-OH-D) was not identified in high amount in most of the reactions with either vitamin D_2_ or D_3_, these bioactive compounds are easily accessible starting from the corresponding calcidiols (1-OH-D), as previously described with *Aae*UPO [49]. In this case, both calcitriols were successfully prepared in near quantitative conversion using *Hsp*UPO and calcidiols as substrates (Fig. 5 and S3).

### Scale-up of 25-hydroxyvitamin D_2_ and D_3_ synthesis with *Hsp*UPO

One of the main drawbacks for the industrial upscaling of the bio-catalyzed hydroxylation of vitamin D (enzymatic as well as whole-cell transformations) is related to the substrate loading. In fact, to the best of our knowledge there are only two approaches of upscaling the hydroxylation of both forms of vitamin D with UPOs. Using r*Aae*UPO (or variants), Huang et al*.* [31] reported excellent conversion and regioselectivity in the hydroxylation of D_3_ and moderate regioselectivity in the case of D_2_ but with low substrate loading (5 and 2 mM, respectively) despite scaling-up the reaction volume. Moreover, Xu and coworkers [30] tried to circumvent the moderate regioselectivity of r*Aae*UPO with the regioselective hydroxylation of 7-dehydrocholesterol followed by a photocatalyzed conversion (to pre-vitamin D_3_) and a subsequent thermal isomerization to 25-OH-D_3_. However, this creative approach depleted the overall yield.

On the other hand, to accomplish efficient and economically feasible application of UPOs as industrial biocatalysts, sufficient amounts of high-quality recombinant enzyme need to be available, making production host as well as specific enzyme sequence a critical choice. In this sense, *K. phaffii* has not only proven to be an excellent host for rUPO production [38], but is also a widespread “industrial workhorse” for the production of recombinant proteins and industrial enzymes, making the technology and expertise for scaling-up protein production readily available. Regarding enzyme choice, especially in combination with producibility, the application of short UPOs is more favorable, as recombinant production yields are typically higher [37, 40].

In this context, aimed at improving the previous results for scaling-up the hydroxylation of vitamin D, several reaction parameters such as cosolvent, solubilizing additive, enzyme dose, and reaction time were optimized to achieve high conversions and regioselectivity. The short UPO *Hsp*UPO was selected for both, good conversion and regioselectivity in the hydroxylation of D_2_ and D_3_, and the previously detailed general robustness and straightforward recombinant production (*Hsp*UPO was produced at around 0.2 g/L in a standard not optimized bioprocess [42]) ‒ especially compared to previously applied UPOs like r*Aae*UPO. Two approaches using different UPO cosubstrates were investigated as described below.

#### Scale-up using peroxide

In preliminary experiments with D_2_, we started to increase the substrate loading from 0.05 mM to 1 mM, using higher concentration of acetone (50% v/v, Table S4), since acetone had been proved to be one of the best organic solvents in terms of UPO stability, and besides, it is not substrate for UPOs [50]. However, the solubility of D_2_ decreased at concentration above 1 mM so we opted to investigate β-cyclodextrins, namely, methyl-(MCD), 2-hydroxypropyl (HPCD) and sulfobutyl-β-cyclodextrin (SBECD), as solubilizing agents to improve the D_2_ solubilization. First, we tested MCD whose addition allow us to dissolve 1 mM of D_2_ using only 20% acetone, and better conversion degrees were observed (up to 70% conversion, Table S4, *entry 5*) within 4 h, as the UPO dose increased. Besides, longer reaction times, up to 24 h and the split dosage of the UPO had a positive effect showing similar conversions to those of the screening and using the same enzyme/substrate ratio (89% conversion, Table S4, *entry 7*). We also explored the use of HPCD as an alternative to MCD but no conversion of vitamin D was produced.

Being aware of the high acetone concentration used and the potential risk of explosion of certain combinations of acetone and hydrogen peroxide[51], we explored the use of *iso*-propanol (^*i*^PrOH) as cosolvent (Table 2). In this sense, using only 1% ^*i*^PrOH in the presence of 10% MCD, the reactions at 5 mM of D_2_ worked with high conversions (86%, Table 2, *entry 2*). It should be noted that this conversion was achieved even using the same UPO dose than 1 mM reactions (80 µM UPO, 8 mol% UPO) representing a fivefold reduction of enzyme (1.6 mol% UPO). Then, we investigated the use of HPCD and SBECD as solubilizing agents, but the D_2_ conversions depleted dramatically (to 13% and 10%, respectively, *entries 3 and 4*).

**Table 2:** Scale-up of vitamins D (D_2_ and D_3_) reactions (24 h) with *Hsp*UPO using peroxide as cosubstrate under different conditions, showing the 25-hydroxyvitamin D (%) formed that in all cases matches with substrate conversion (best conditions and results are highlighted in bold)

Entry	Vitamin D	*Hsp*UPO	H_2_O_2_	Cosolvent	Additive	25-OH-D
1	D_2_, 1 mM	2 × 40 µM	50 mM	Acetone, 20%	MCD, 10%	95%
2	D_2_, 5 mM	2 × 40 µM	50 mM	^*i*^PrOH, 1%	MCD, 10%	86%
3	D_2_, 5 mM	2 × 40 µM	50 mM	^*i*^PrOH, 1%	HPCD, 10%	13%
4	D_2_, 5 mM	2 × 40 µM	50 mM	^*i*^PrOH, 1%	SBECD, 10%	10%
5	D_2_, 10 mM	2 × 40 µM	50 mM	^*i*^PrOH, 1%	MCD, 10%	48%
6	D_2_, 10 mM	2 × 40 µM	100 mM	^*i*^PrOH, 1%	MCD, 10%	49%
7	D_2_, 10 mM	2 × 60 µM	50 mM	^*i*^PrOH, 1%	MCD, 10%	76%
8	D_2_, 10 mM	2 × 60 µM	100 mM	^*i*^PrOH, 1%	MCD, 10%	85%
9	D_2_, 15 mM	2 × 60 µM	50 mM	^*i*^PrOH, 1%	MCD, 10%	50%
10	D_2_, 15 mM	2 × 60 µM	100 mM	^*i*^PrOH, 1%	MCD, 10%	42%
11	D_2_, 15 mM	2 × 80 µM	50 mM	^*i*^PrOH, 1%	MCD, 10%	70%
12	D_2_, 15 mM	2 × 80 µM	100 mM	^*i*^PrOH, 1%	MCD, 10%	58%
13	D_2_, 15 mM	2 × 80 µM	50 mM	^*i*^PrOH, 2%	MCD, 20%	70%
14	D_2_, 15 mM	2 × 80 µM	100 mM	^*i*^PrOH, 2%	MCD, 20%	86%
**15**	**D**_**2**_**, 20 mM**	**2 × 80 µM**	**100 mM**	^***i***^**PrOH, 2%**	**MCD, 20%**	**88%**
16	D_2_, 20 mM	2 × 80 µM	100 mM	^*i*^PrOH, 3%	MCD, 30%	79%
17	D_3_, 15 mM	2 × 80 µM	100 mM	^*i*^PrOH, 2%	MCD, 20%	75%
18	D_3_, 20 mM	2 × 80 µM	100 mM	^*i*^PrOH, 2%	MCD, 20%	40%
19	D_3_, 20 mM	2 × 80 µM	150 mM	^*i*^PrOH, 2%	MCD, 20%	30%
20	D_3_, 20 mM	2 × 100 µM	100 mM	^*i*^PrOH, 2%	MCD, 20%	40%
21	D_3_, 20 mM	2 × 80 µM	100 mM	^*i*^PrOH, 3%	MCD, 30%	41%
**22**	**D**_**3**_**, 20 mM**	**2 × 100 µM**	**100 mM**	^***i***^**PrOH, 3%**	**MCD, 30%**	**63%**

Next, using again MCD (10%) under different conditions (*entries 5–8*) the reaction was successfully upscaled to 10 mM D_2_ loading after increasing the UPO and peroxide concentrations to 120 µM and 100 mM, respectively, achieving 85% of both D_2_ conversion and 25-OH-D_2_ formation.

In reactions at 15 mM D_2_, the conversions decreased in the presence of 10% MCD and 1% ^*i*^PrOH even when using higher UPO doses (50%–70% conversion, *entries 9–12*). These results were improved using 20% MCD, 2% ^*i*^PrOH and 160 µM UPO, and 86% D_2_ conversion (5.32 g/L of 25-OH-D_2_, *entry 14*) was attained. These results evidenced the importance of D_2_ solubilization for a successful reaction. Moreover, we also tested D_3_ hydroxylation under the same conditions, and 75% conversion (4.51 g/L of 25-OH-D_3_, *entry 17*) was achieved.

Finally, we tried to optimize the scaling-up of both reactions increasing the substrate (D_2_ and D_3_) loading up to 20 mM, and different results between D_2_ and D_3_ were obtained. On one hand, D_2_ was converted with high yield (88%), while maintaining the same MCD, UPO, and peroxide concentrations as in 15 mM reactions (7.26 g/L of 25-OH-D_2_, *entry 15*). This represents a tenfold reduction of UPO dose (0.8 mol% per mol of D_2_) compared with the screening conditions. Besides, the increasing of MCD and ^*i*^PrOH did not improve the conversion, producing a slight reduction to 79% (*entry 16*). On the other hand, a significant decline in substrate conversion was observed in D_3_ reactions (*entries 18–21*) that only was partially mitigated by the use of higher concentrations of UPO and solubilizing agents (200 µM UPO, 30% MCD, and 3% ^*i*^PrOH, *entry 22*) obtaining 63% 25-OH-D_3_ (5.04 g/L).

#### Scale-up using O_2_/reductants

**Fig. 6:**
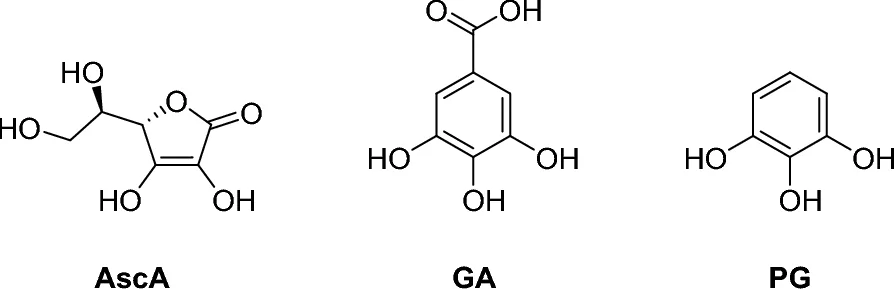
Reductants used with O_2_ as UPO cosubstrates: ascorbic acid (AscA), gallic acid (GA), and pyrogallol (PG)

Aimed to improve or provide an alternative for scaling-up the synthesis of 25-OH-D, we decided to use a different UPO-activation procedure, using oxygen and certain reductants-vicinal diols such as ascorbic acid (AscA), gallic acid (GA), and pyrogallol (PG) (Fig. 6), based on the very recent work by Deng et al*.* [52]. These compounds can be easily obtained from plant biomass sources [53–55]. This is the first time that this procedure is used in a UPO reaction with vitamins D (Table 3).

**Table 3:** Scale-up of vitamins D (D_2_ and D_3_) reactions with *Hsp*UPO using O_2_/reductants as cosubstrates under different conditions, showing the 25-hydroxyvitamin D (%) formed that in all cases matches with substrate conversion (best conditions and results are highlighted in bold)

Entry	Vitamin D	Reductant	Time	25-OH vitamin D
1	D_2_, 15 mM	AscA, 15 mM	24 h	52%
2	D_2_, 15 mM	AscA, 15 mM	48 h	72%
3	D_2_, 20 mM	AscA, 20 mM	72 h	81%
4	D_2_, 20 mM	GA, 20 mM	72 h	68%
5	D_2_, 20 mM	PG, 20 mM	24 h	86%
**6**	**D**_**2**_**, 20 mM**	**PG, 20 mM**	**48 h**	**97%**
7	D_2_, 20 mM	PG, 20 mM	72 h	95%
8	D_3_, 15 mM	AscA, 15 mM	72 h	75%
9	D_3_, 20 mM	AscA, 20 mM	72 h	65%
10	D_3_, 20 mM	GA, 20 mM	72 h	67%
**11**	**D**_**3**_**, 20 mM**	**PG, 20 mM**	**72 h**	**75%**

Reaction conditions: *Hsp*UPO 160 µM (single dose), MCD 20% (w/v) and 2% ^*i*^PrOH

The experiments were conducted with 160 µM of *Hsp*UPO (added in a single dose) in the presence of 20% MCD and 2% ^*i*^PrOH (Table 3). The first reactions with D_2_ were carried out at 15 mM substrate loading using AscA as reductant, for 24 h. Different concentrations (5–60 mM) of AscA were tested (data not shown), and the highest conversion (52%) was achieved with 15 mM AscA, that is the same concentration as the substrate (*entry 1*). In fact, as proposed by Deng et al*.*, since the reductant helps in the UPO-activation, the lower concentration of the reductant than the substrate is compatible with a lower substrate transformation, although the negative effect of the higher concentration of reductant is unclear. Next, the reaction time was increased up to 48 h, and a significant increase in substrate conversion (up to 72%) was produced (*entry 2*). When 20 mM D_2_ with 20 mM AscA was tested, up to 81% conversion was attained within 72 h (*entry 3*).

At this point, the reductants GA and PG were also investigated with 20 mM of D_2_. Under the optimized conditions, a moderate conversion of 68% was produced with GA after 72 h (*entry 4*). Interestingly, remarkable 86% and 97% D_2_ conversions were achieved with PG after 24 h and 48 h, respectively (*entries 5–6*).

On the other hand, the reaction of 15 mM D_3_ with AscA delivered 75% conversion under the optimized conditions (*entry 8*), matching the result reached with H_2_O_2_ (Table 2, *entry 17*). In 20 mM D_3_ reactions using AscA and GA, the conversions (65% and 67%, respectively) decreased (*entries 9–10*), in contrast with the one using PG, with 75% D_3_ conversion, which represents the highest production of 25-OH-D_3_ (5.37 g/L) achieved with UPOs.

In contrast to D_2_, there is a remarkable decrease of D_3_ productivity in a dose dependent manner with both procedures (peroxides and reductants). This fact could be explained by the apparent decrease in D_3_ solubility as the substrate loading increased.

Despite that the overall results in terms of yield and potential isolated product amount are in very good range, especially with the D_2_ form (19.4 mM, 8 g/L, 25-OH-D_2_), the total turnover number (TTN) obtained (TTN = 121) is relatively low due to the high charge of enzyme required. Further process engineering and improved enzyme stability may substantially increase the TTN of this process in the future.

## Conclusions

A comprehensive study on the regioselective C25-hydroxylation of vitamins D_2_ and D_3_ has been performed using a series of 71 UPOs, recombinantly produced with *K. phaffii*. Overall, the reactions with D_2_ resulted in the formation of the 25-hydroxy derivative with higher regioselectivity than with D_3_. Particularly, 42 UPOs out of 71 were able to convert D_2_ with strict/high regioselectivity and high degrees of conversion (up to 99%) to 25-OH-D_2_. Among these, the 16 best UPOs were tested in D_3_ reactions where they showed less regioselectivity than with D_2_, since in addition to the desired 25-hydroxy derivative they produced other di- and tri-hydroxylated products, the only exception being *Hsp*UPO. Finally, the scale-up of both reactions was conducted using *Hsp*UPO ‒ because of its favorable regioselectivity with both vitamins and the previously reported ease of recombinant production and general robustness – and MCD plus minor amounts of ^*i*^PrOH as solubilizing agents. Four different cosubstrates, namely, H_2_O_2_ and O_2_ with AscA, GA, or PG were investigated in the upscaling, and very good product yields and strict regioselectivity were attained in all cases (up to 19 mM for vitamin D_2_ and 15 mM for D_3_) especially with the latter cosubstrate. The new procedure using O_2_/reductants takes advantage of avoiding the UPO-inactivation by H_2_O_2_, and the need to produce engineered variants with high tolerance to them. Finally, the relatively low TTN attained still has room for improvement and investigations in process engineering may improve catalytic efficiency in the near future.

## Supplementary Information

Supplementary Material 1. GC-MS chromatograms of D_2_ and D_3_ reactions with UPOs, GC-MS chromatograms of calcidiol and calcitriol standards of D_2_ and D_3_, *Hsp*UPO reactions with calcidiols (D_2_ and D_3_), Table of UPO accession numbers and other details for protein production, Tables of UPO reactions with D_2_ and D_3_ and Table of preliminary upscaling of D_2_ reaction

## Data Availability

The datasets used and/or analyzed during the current study are available from the corresponding author on reasonable request.
