# Supplementary material for: Comprehensive assessment of fungal peroxygenases for the selective synthesis of 25-hydroxyvitamins D2 and D3
Source: Biotechnol Biofuels Bioprod. 2026 Jul 10;19:76. doi: 10.1186/s13068-026-02792-0 (PMC13640091; doi:10.1186/s13068-026-02792-0)
Supplement: Supplementary file 1 [file 13068_2026_2792_MOESM1_ESM.pdf]

Supplementary Information

Comprehensive assessment of fungal peroxygenases for the selective synthesis of 25-hydroxyvitamins D_2 and D_3

Alejandro González-Benjumea‡[a], Andrés Olmedo‡ [a], Katharina Ebner[b], Anton Glieder[b], and Ana Gutiérrez[a]*

[a] Instituto de Recursos Naturales y Agrobiología de Sevilla (IRNAS), CSIC, Reina Mercedes 10, E-41012 Sevilla, Spain

[b] Bisy GmbH, Hofstätten an der Raab, Austria

[+] These authors contributed equally to this work

[*] Corresponding author

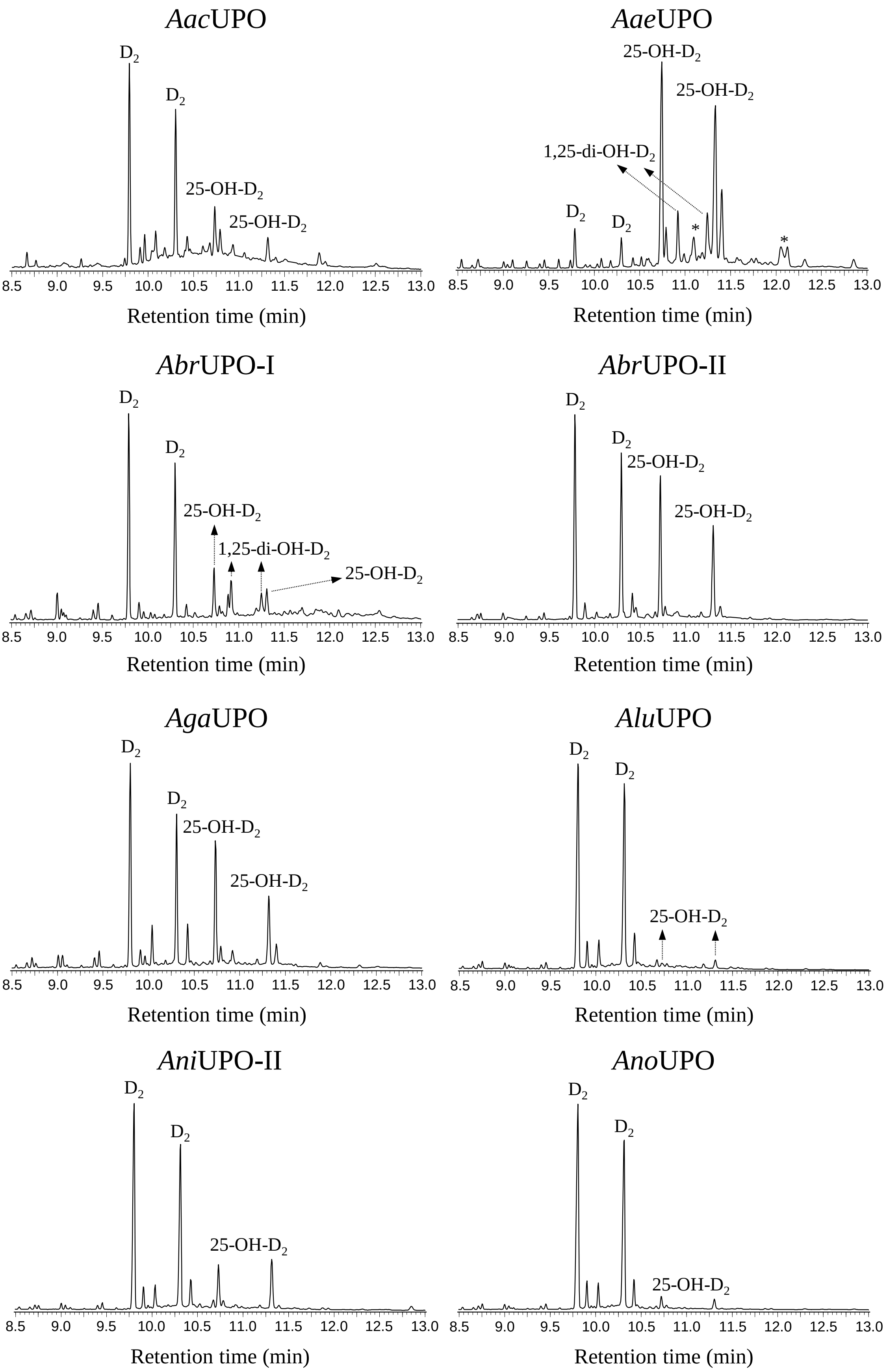

Fig. S1 GC-MS chromatograms of vitamin D_2 reactions with different UPOs. *Unidentified products

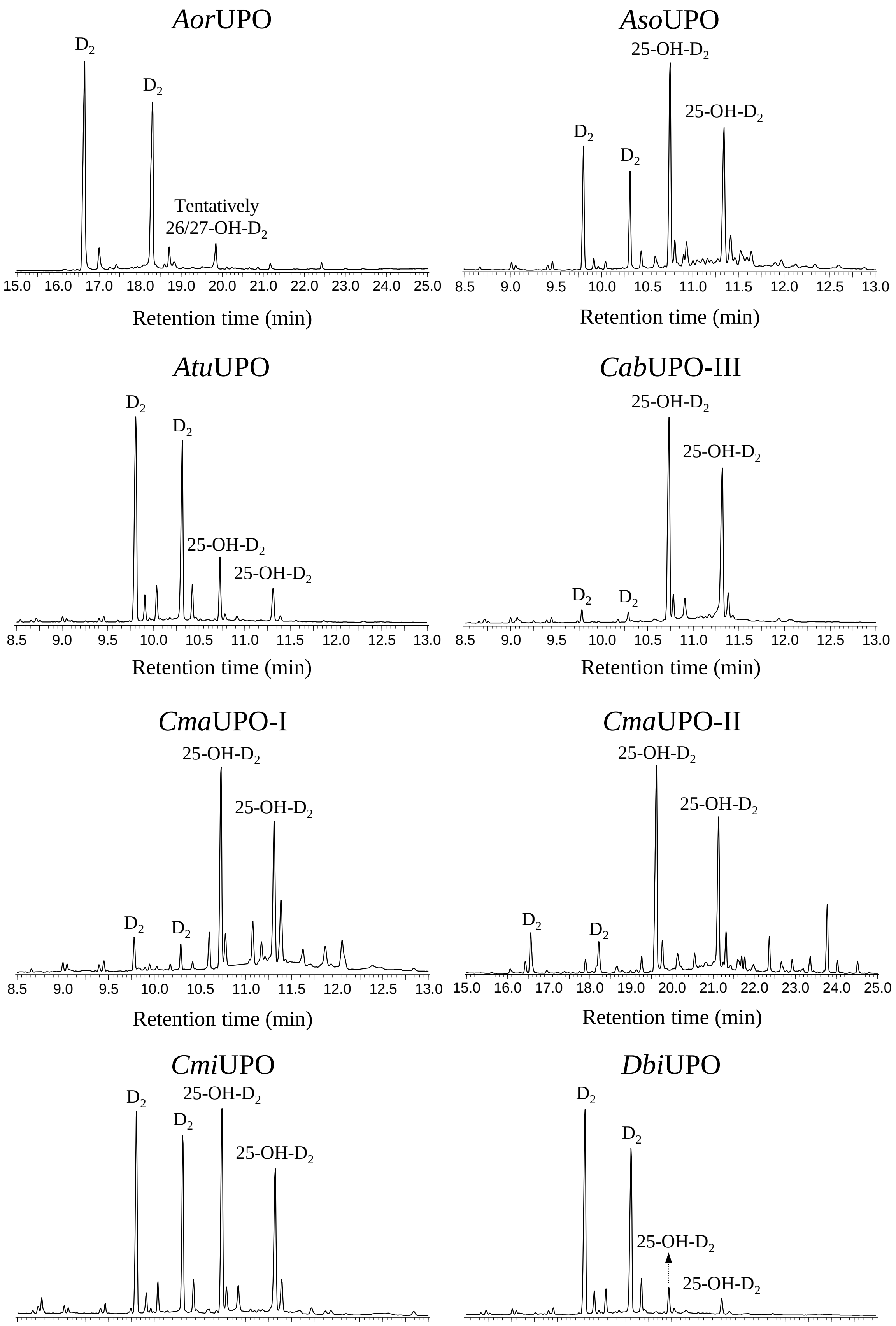

*Aor*UPO
D2
D2
Tentatively
26/27-OH-D2
15.0 16.0 17.0 18.0 19.0 20.0 21.0 22.0 23.0 24.0 25.0
Retention time (min)
*Aso*UPO
25-OH-D2
25-OH-D2
D2
D2
8.5 9.0 9.5 10.0 10.5 11.0 11.5 12.0 12.5 13.0
Retention time (min)
*Atu*UPO
D2
D2
25-OH-D2
25-OH-D2
8.5 9.0 9.5 10.0 10.5 11.0 11.5 12.0 12.5 13.0
Retention time (min)
*Cab*UPO-III
25-OH-D2
25-OH-D2
D2
D2
8.5 9.0 9.5 10.0 10.5 11.0 11.5 12.0 12.5 13.0
Retention time (min)
*Cma*UPO-I
25-OH-D2
25-OH-D2
D2
D2
8.5 9.0 9.5 10.0 10.5 11.0 11.5 12.0 12.5 13.0
Retention time (min)
*Cma*UPO-II
25-OH-D2
25-OH-D2
D2
D2
15.0 16.0 17.0 18.0 19.0 20.0 21.0 22.0 23.0 24.0 25.0
Retention time (min)
*Cmi*UPO
D2
25-OH-D2
D2
25-OH-D2
8.5 9.0 9.5 10.0 10.5 11.0 11.5 12.0 12.5 13.0
Retention time (min)
*Dbi*UPO
D2
D2
25-OH-D2
25-OH-D2
8.5 9.0 9.5 10.0 10.5 11.0 11.5 12.0 12.5 13.0
Retention time (min)

Fig. S1 (cont.)

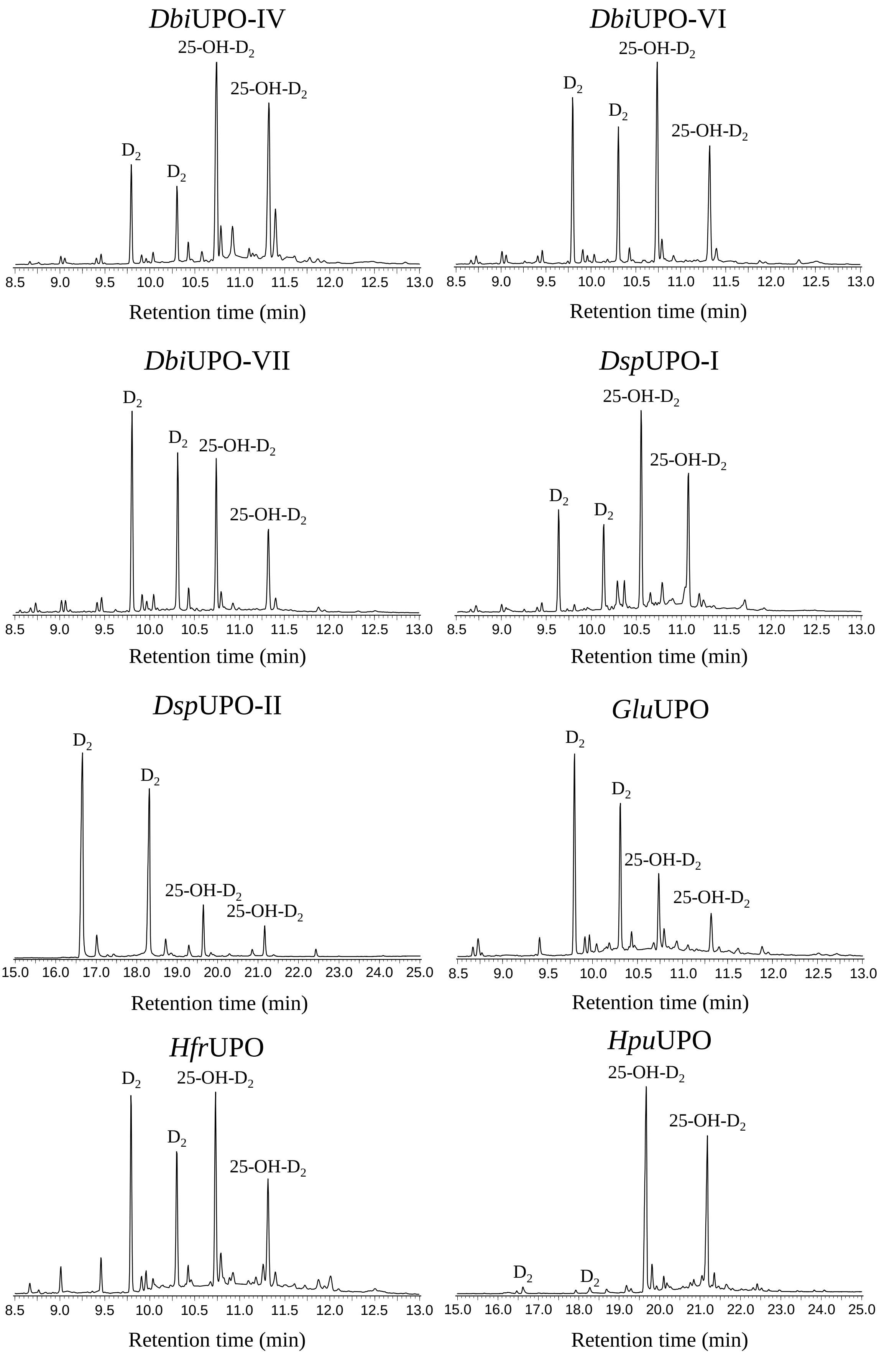

DbiUPO-IV
D2
D2
25-OH-D2
25-OH-D2
Retention time (min)
DbiUPO-VI
D2
D2
25-OH-D2
25-OH-D2
Retention time (min)
DbiUPO-VII
D2
D2
25-OH-D2
25-OH-D2
Retention time (min)
DspUPO-I
D2
D2
25-OH-D2
25-OH-D2
Retention time (min)
DspUPO-II
D2
D2
25-OH-D2
25-OH-D2
Retention time (min)
GluUPO
D2
D2
25-OH-D2
25-OH-D2
Retention time (min)
HfrUPO
D2
D2
25-OH-D2
25-OH-D2
Retention time (min)
HpuUPO
D2
D2
25-OH-D2
25-OH-D2
Retention time (min)

Fig. S1 (cont.)

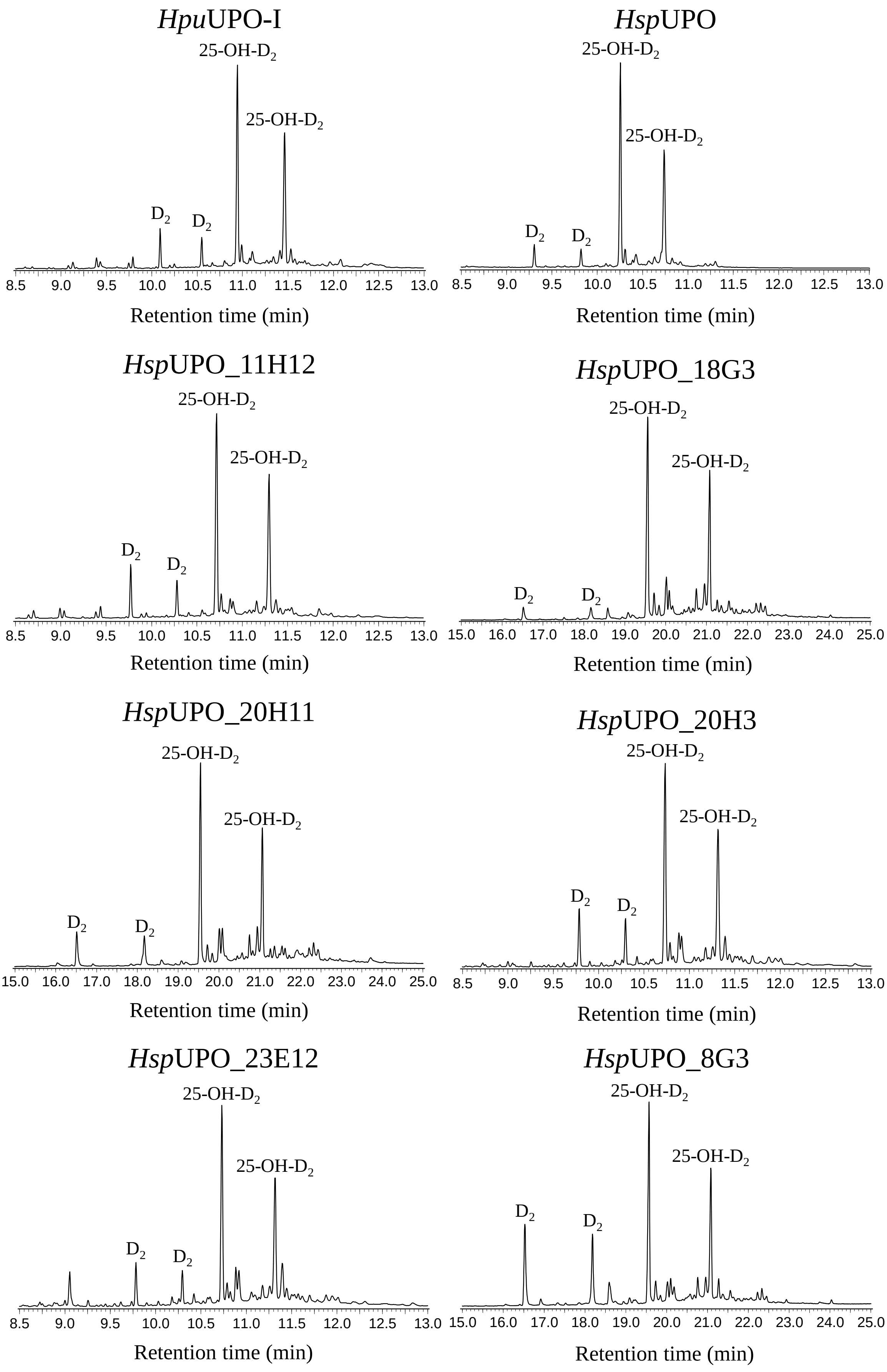

*Hpu*UPO-I
25-OH-D_2
25-OH-D_2
D_2
D_2
8.5 9.0 9.5 10.0 10.5 11.0 11.5 12.0 12.5 13.0
Retention time (min)
*Hsp*UPO
25-OH-D_2
25-OH-D_2
D_2
D_2
8.5 9.0 9.5 10.0 10.5 11.0 11.5 12.0 12.5 13.0
Retention time (min)
*Hsp*UPO_11H12
25-OH-D_2
25-OH-D_2
D_2
D_2
8.5 9.0 9.5 10.0 10.5 11.0 11.5 12.0 12.5 13.0
Retention time (min)
*Hsp*UPO_18G3
25-OH-D_2
25-OH-D_2
D_2
D_2
15.0 16.0 17.0 18.0 19.0 20.0 21.0 22.0 23.0 24.0 25.0
Retention time (min)
*Hsp*UPO_20H11
25-OH-D_2
25-OH-D_2
D_2
D_2
15.0 16.0 17.0 18.0 19.0 20.0 21.0 22.0 23.0 24.0 25.0
Retention time (min)
*Hsp*UPO_20H3
25-OH-D_2
25-OH-D_2
D_2
D_2
8.5 9.0 9.5 10.0 10.5 11.0 11.5 12.0 12.5 13.0
Retention time (min)
*Hsp*UPO_23E12
25-OH-D_2
25-OH-D_2
D_2
D_2
8.5 9.0 9.5 10.0 10.5 11.0 11.5 12.0 12.5 13.0
Retention time (min)
*Hsp*UPO_8G3
25-OH-D_2
25-OH-D_2
D_2
D_2
15.0 16.0 17.0 18.0 19.0 20.0 21.0 22.0 23.0 24.0 25.0
Retention time (min)

Fig. S1 (cont.)

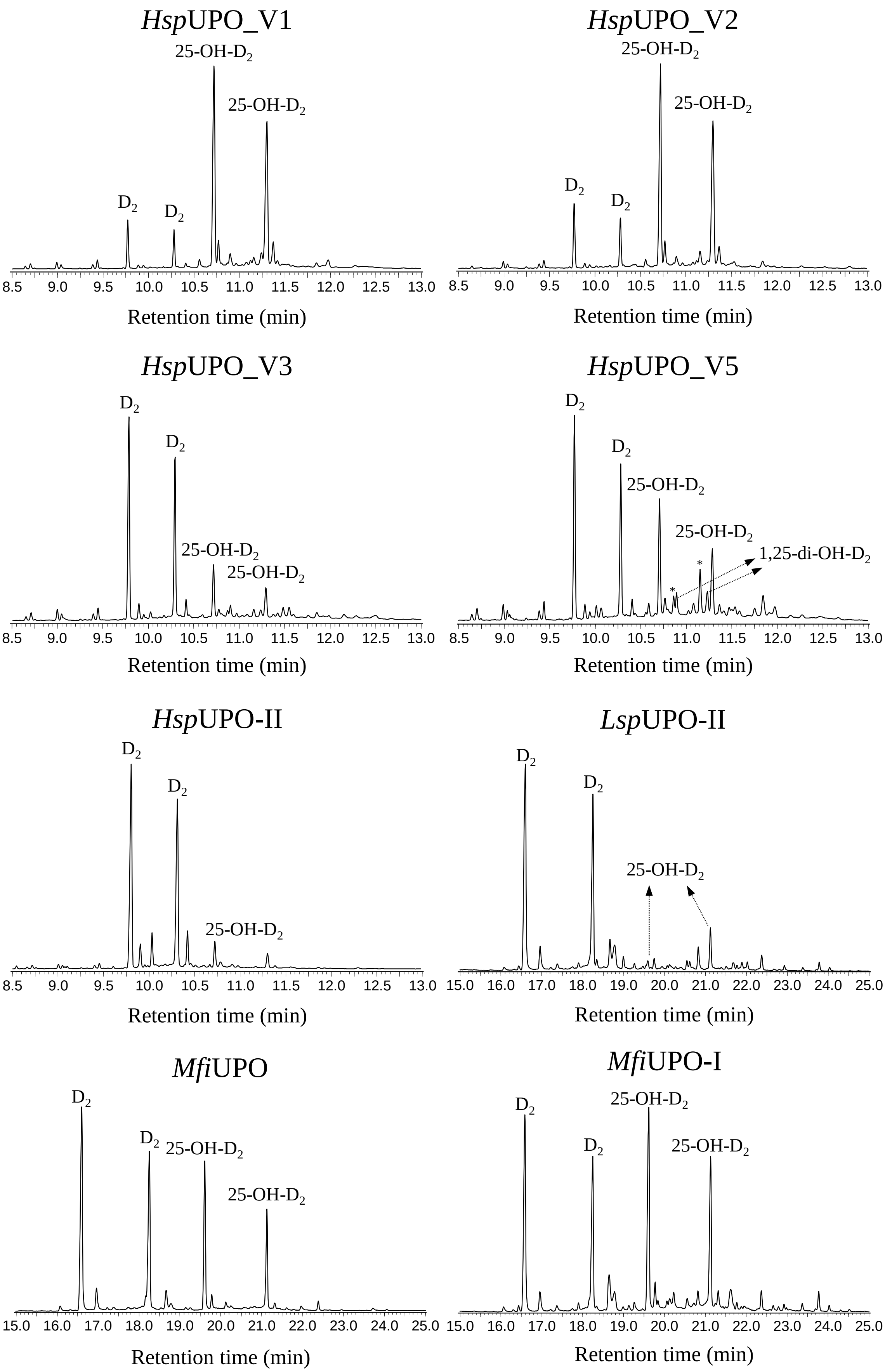

Fig. S1 (cont.) *Unidentified products.

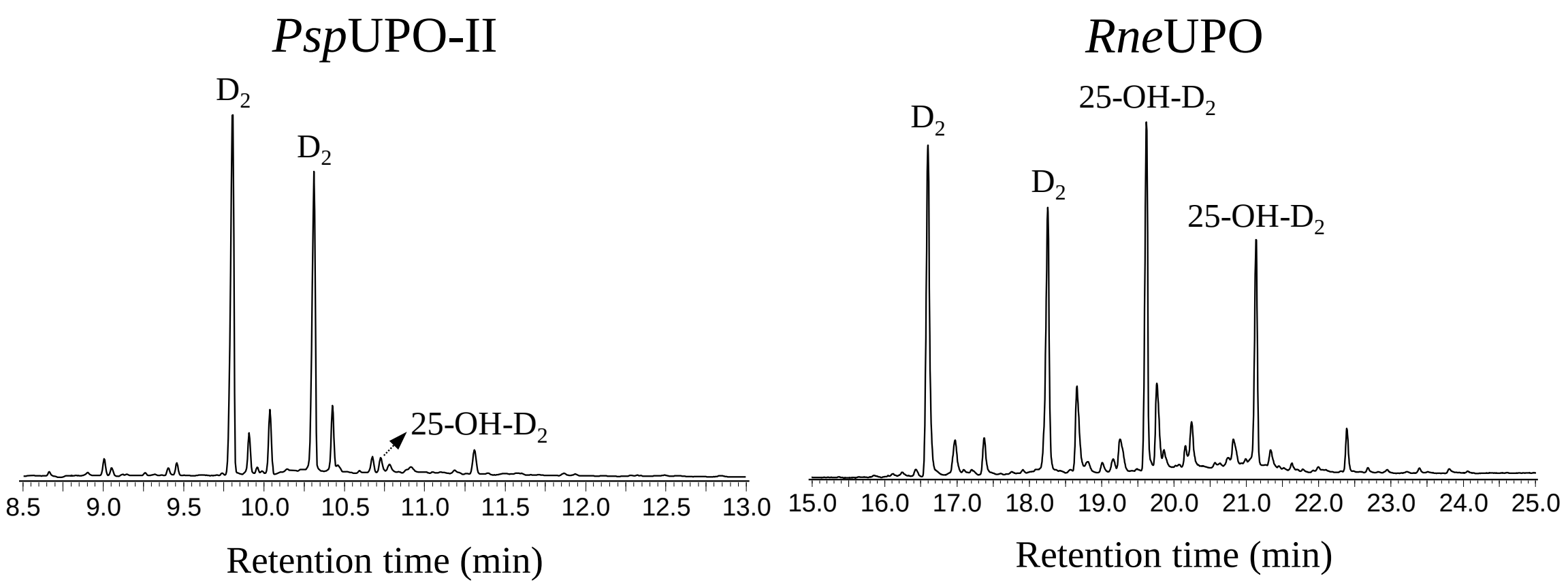

*Psp*UPO-II
D2
D2
25-OH-D2
8.5
9.0
9.5
10.0
10.5
11.0
11.5
12.0
12.5
13.0
Retention time (min)
*Rne*UPO
D2
25-OH-D2
D2
25-OH-D2
15.0
16.0
17.0
18.0
19.0
20.0
21.0
22.0
23.0
24.0
25.0
Retention time (min)

Fig. S1 (cont.)

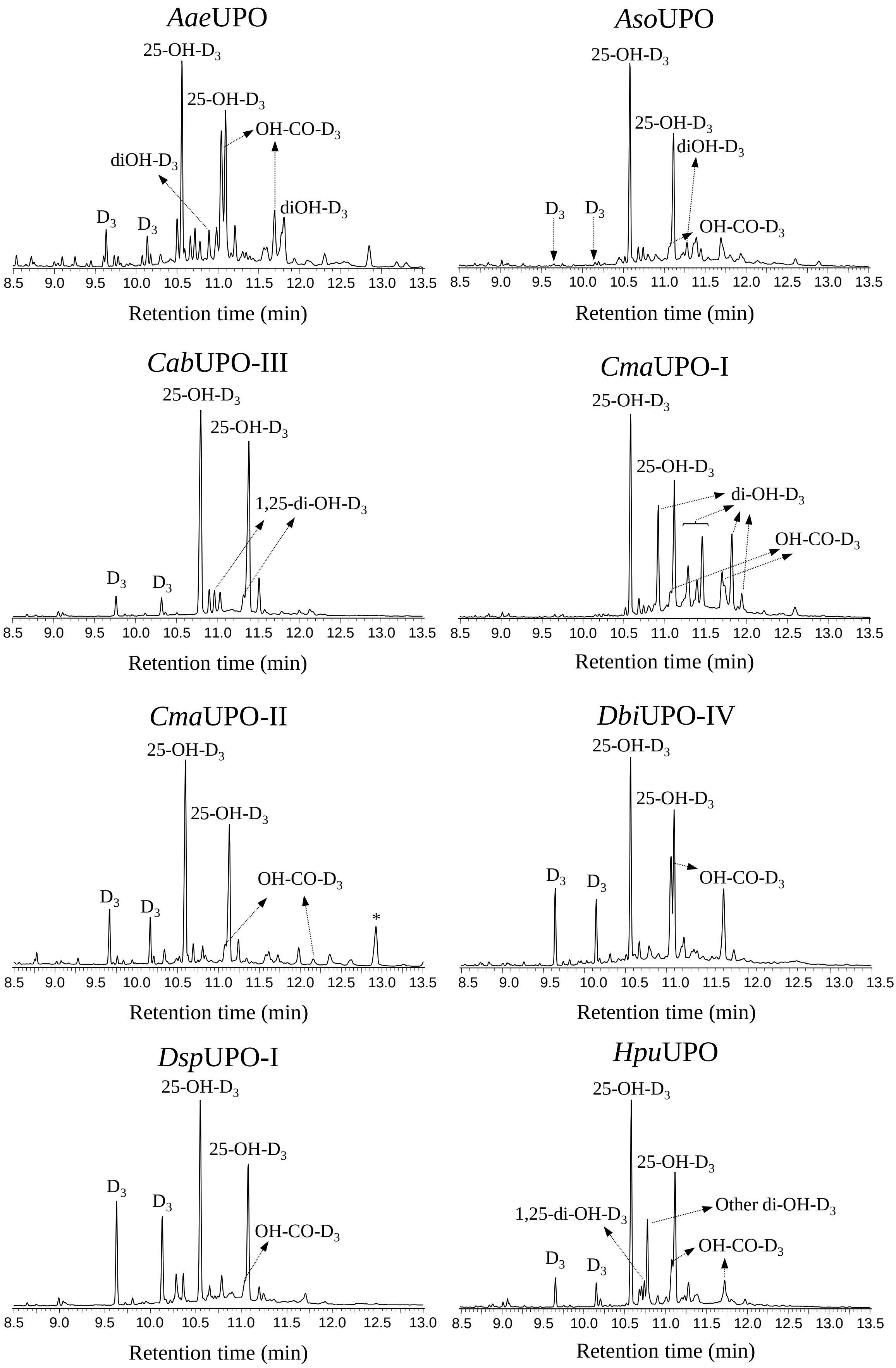

Fig. S2 GC-MS chromatograms of vitamin D_3 reactions with different UPOs. *Unidentified products

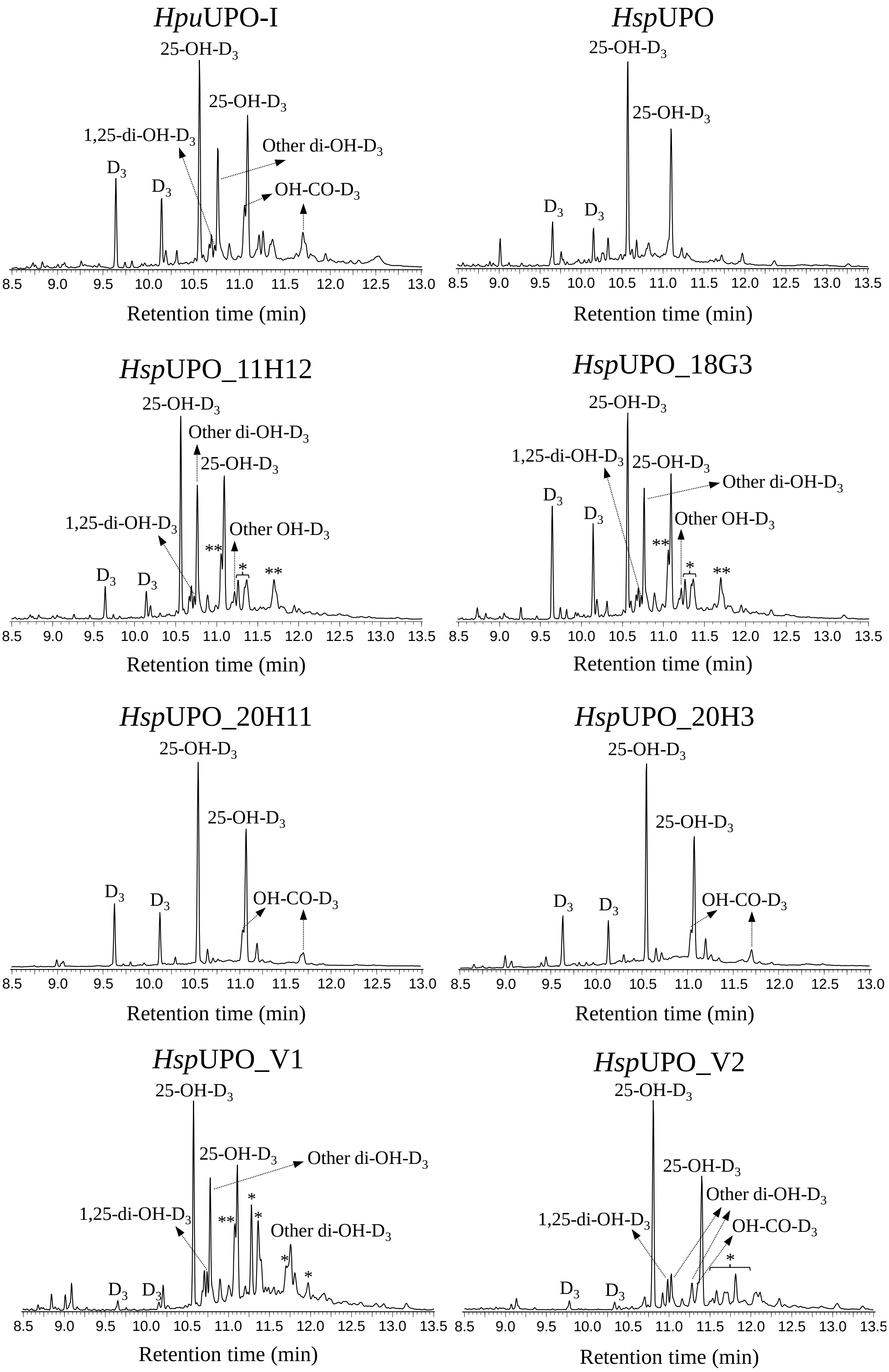

Fig. S2 (cont.) *Unidentified products. **OH-CO-D_3

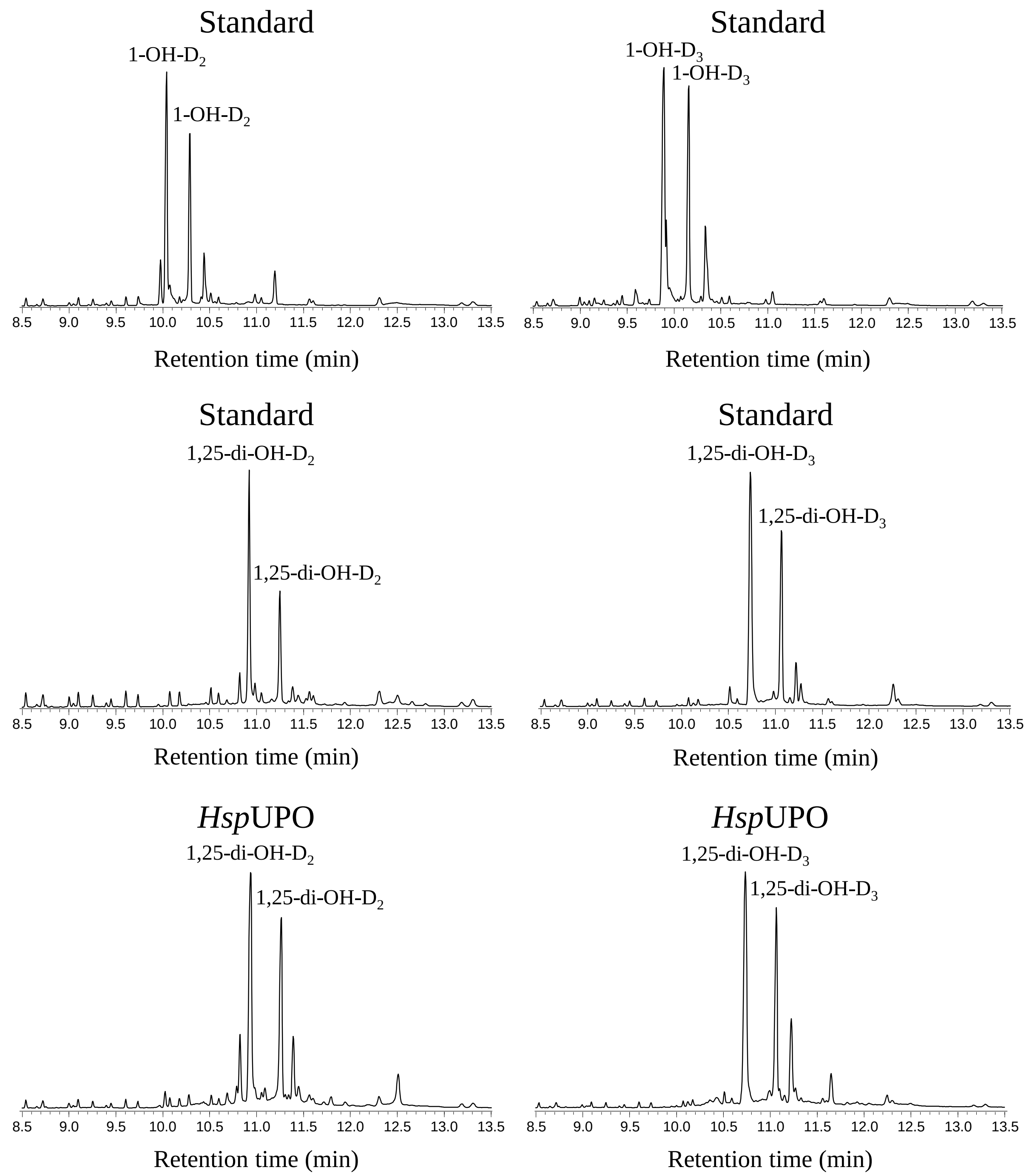

Fig. S3 Calcidiol standards (*top*), calcitriol standards (*middle*) and *Hsp*UPO reactions with calcidiols producing calcitriols (*bottom*). D_2 form, *left*; D_3 form, *right*

Table S1 UPOs used in this study not previously described

**Abbreviation**	**NCBI accession number**	**Organism of origin**	**Protein sequence (if no Accession number available) or mutation**	**Division**	**Long/ Short UPO**
		**Wild-type UPOs**			
*Aor*UPO	OOO07519.1	*Aspergillus oryzae*		Ascomycota	S
*Hpu*UPO	-	*Hypoxylon pulicicidum*	MRFPSIFTAVLFAASS<br>ALAAPVNTTTEDETA<br>QIPAEAVIGYSDLEGD<br>FDVAVLPFSASIAAKE<br>EGVSLEKREAEAAPS<br>SSGWEAPGPNDVRAP<br>CPMLNTLANHGFLPH<br>SGKGITVNKTIDALGS<br>ALNINANLSTLLFSFA<br>ATTNPQPNATFFDLD<br>HLSRHNILEHDASLSR<br>QDSYFGPADVFNEAV<br>FNQTKSFWTGDIIDV<br>QMAANARVVRLLTS<br>NLTNPEYSLSDLGSAF<br>SAGESAAYIGILGDKK<br>TATVPKTWVEYLFEN<br>ERLPYELGFKKPADPF<br>TQDDLSDLSLKIIDSQ<br>HFPPQSPGKVSKRDV<br>HCPYGFH*		
*Dbi*UPO-VI	THU90505.1	*Dendrothele bispora*			
*Hfr*UPO	-	*Hypoxylon fragiforme*	MRFPSIFTAVLFAASS<br>ALAAPVNTTTEDETA<br>QIPAEAVIGYSDLEGD<br>FDVAVLPFSASIAAKE<br>EGVSLEKREAEAASL<br>PGWAPAGPNDVRAP<br>CPMLNALANHGYLP<br>HDGKGITVNKTVDAL<br>KSALNFDPVLSSSLFE<br>FAATTNPIPNATFFDL<br>DHLSRHNILEHDASLS<br>RQDAYFGRADTFNEA<br>VFNQTKSYWTGDIIT<br>VQMASNARVSRLMT<br>SNLTNPEYSLSDLGSE<br>FSIGETAAYISVLGDK<br>KTGTVPKSYVEFLFE<br>KERLPWELGFKRAAE<br>PFTEQDLADMMGQIV<br>NAQHFPQEPGKVGW*		
*Hpu*UPO-I	-	*Hypoxylon pulicicidum*	MRFPSIFTAVLFAASS<br>ALAAPVNTTTEDETA<br>QIPAEAVIGYSDLEGD		

			FDVAVLPFSASIAAKE<br>EGVSLEKREAEAAPS<br>SSGWEAPGPNDVRAP<br>CPMLNTLANHGFLPH<br>SGKGITVNKTIDALGS<br>ALNINANLSTLLFSFA<br>ATTNPQPNATFFDLD<br>HLSRHNILEHDASLSR<br>QDSYFGPADVFNEAV<br>FNQTKSFWTGDIIDV<br>QMAANARVVRLLTS<br>NLTNPEYSLSDLGSAF<br>SVGESAAYIGILGDKK<br>TATVPKTWVEYLFEN<br>ERLPYELGFKKPADPF<br>TQDDLSDLSLKIIDAQ<br>HFPPQSPGKVSKRDIH<br>CPYGFH*		
***Hsp*UPO mutants**					
*Hsp*UPO_V1	7O1R_A	*Hypoxylon sp.* EC38	L247F	Ascomycota	S
*Hsp*UPO_V2	7O1R_A	*Hypoxylon sp.* EC38	L247H		
*HHsp*UPO_V4	7O1R_A	*Hypoxylon sp.* EC38	L247M		
*Hsp*UPO_23E12	7O1R_A	*Hypoxylon sp.* EC38	S24F		
*Hsp*UPO_8G3	7O1R_A	*Hypoxylon sp.* EC38	C256S		
*Hsp*UPO_18G3	7O1R_A	*Hypoxylon sp.* EC38	D253I		
*Hsp*UPO_11H12	7O1R_A	*Hypoxylon sp.* EC38	C256X		
*Hsp*UPO_20H3	7O1R_A	*Hypoxylon sp.* EC38	R251L		
*Hsp*UPO_20H11	7O1R_A	*Hypoxylon sp.* EC38	E249X		

Table S2 Substrate (D_2) conversion by several UPOs and relative percentage of products

UPO	25-OH-D_2 (%)	X-OH-D_2 (%)	Conversion (%)
*Aac*UPO	20	-	20
*Aae*UPO	62	16[a]	92[b]
*Abo*UPO	-	-	-
*Abr*UPO-I	16	13[a]	29
*Abr*UPO-II	40	-	40
*Ael*UPO	-	-	-
*Aga*UPO	37	-	37
*Alu*UPO	-	-	-
*Ani*UPO-II	20	-	20
*Ano*UPO	4	-	4
*Aor*UPO	-	5[c]	5
*Aps*UPO	-	-	-
*Aso*UPO	70	-	70
*Atu*UPO	17	-	17
*Cab*UPO-III	91	5[a]	96
*Cab*UPO-IV	-	-	-
*Cab*UPO-V	-	-	-
*Cab*UPO-VI	-	-	-
*Cab*UPO-VII	-	-	-
*Cma*UPO-I	88	-	88
*Cma*UPO-II	86	-	86
*Cmi*UPO	52	-	52
*Dbi*UPO	8	-	8
*Dbi*UPO-IV	73	-	73
*Dbi*UPO-V	-	-	-
*Dbi*UPO-VI	56	-	56
*Dbi*UPO-VII	41	-	41
*Dsp*UPO-I	84	-	84
*Dsp*UPO-II	6	-	6
*Ela*UPO	-	-	-
*Gdi*UPO-I	-	-	-
*Gdi*UPO-II	-	-	-
*Glu*UPO	26	-	26
*Gma*UPO-I	-	-	-
*Gma*UPO-II	-	-	-
*Hfr*UPO	50		50
*Hpu*UPO	>99	-	>99
*Hpu*UPO-I	86	-	86
*Hsp*UPO	90	-	90
*Hsp*UPO_11H12	83	-	83
*Hsp*UPO_18G3	91	-	91
*Hsp*UPO_20H11	82	-	82
*Hsp*UPO_20H3	76	-	76
*Hsp*UPO_23E12	77	-	77
*Hsp*UPO_8G3	69	-	69
*Hsp*UPO_V1	85	-	85

[a]1,25-DiOH-D_2; [b]14% unidentified products; [c]26/27-OH-D_2. Conditions: 0.05 mM D_2; 4 µM UPO; 2.5 mM H_2O_2, 1 h

Table S2 (cont.)

UPO	25-OH-D_2 (%)	X-OH-D_2 (%)	Conversion (%)
*Hsp*UPO_V2	80	-	80
*Hsp*UPO_V3	20	-	20
*Hsp*UPO_V5	30	7[a]	47[d]
*Hsp*UPO-II	16	-	16
*Hwe*UPO	-	-	-
*Lam*UPO	-	-	-
*Lsp*UPO-II	9	-	9
*Mfi*UPO	36	-	36
*Mfi*UPO-I	46	-	46
*Mni*UPO	-	-	-
*Mor*UPO	-	-	-
*Mor*UPO_V1	-	-	-
*Mor*UPO_V4	-	-	-
*Mor*UPO_V5	-	-	-
*Mve*UPO	-	-	-
*Pan*UPO	-	-	-
*Psp*UPO-II	4	-	4
*Rne*UPO	45	-	45
*Sch*UPO	-	-	-
*Sst*UPO-II	-	-	-
*Sst*UPO-III	-	-	-
*Ssu*UPO	-	-	-
*Vno*UPO	-	-	-
*Vvi*UPO	-	-	-
*Xmu*UPO	-	-	-

[a]1,25-DiOH-D_2; [d]10% unidentified products. Conditions: 0.05 mM D_2; 4 µM UPO; 2.5 mM H_2O_2, 1 h.

Table S3 Substrate (D_3) conversion by several UPOs and relative percentage of products

UPO	25-OH-D_3 (%)	OH-D_3 (%)	di-OH-D_3 (%)	OH-CO-D_3 (%)	Conversion (%)
*Aae*UPO	46	-	13[a]	35	94
*Aso*UPO	83	-	5	12	>99
*Cab*UPO-III	87	-	7[b]	-	94
*Cma*UPO-I	37	-	52	11	>99
*Cma*UPO-II	64	-	-	4	84[i]
*Dbi*UPO-IV	48	-	-	35	83
*Dsp*UPO-I	63	-	-	4	67
*Hpu*UPO	59	-	17[c]	16	92
*Hpu*UPO-I	48	-	18[d]	14	80
*Hsp*UPO	84	-	-	-	84
*Hsp*UPO_V1	32	-	32[e]	5	98[j]
*Hsp*UPO_V2	67	-	16[f]	9	98[k]
*Hsp*UPO_18G3	40	2	15[g]	14	78[l]
*Hsp*UPO_20H3	72			9	81
*Hsp*UPO_20H11	69			11	80
*Hsp*UPO_11H12	45	3	21[h]	16	92[m]

1,25-Di-OH-D_3: [a]4%; [b]7%; [c]4%; [d]2%; [e]3%; [f]4%; [g]1%; [h]2%. Unidentified products: [i]16%; [j]29%; [k]6%; [l]7%; [m]7%. Conditions: 0.05 mM D_3; 4 µM UPO; 2.5 mM H_2O_2, 1 h

Table S4 Reactions of 1 mM vitamin D_2 with *Hsp*UPO_18G3 at 50 mM H_2O_2

**Entry**	**UPO doses**	**Acetone (%)**	**Additive**	**Time**	**25-OH-D_2**
1	8 µM	50%	-	4 h	34%
2	16 µM	50%	-	4 h	42%
3	16 µM	20%	MCD, 10%	4 h	47%
4	50 µM	20%	MCD, 10%	4 h	53%
5	80 µM	20%	MCD, 10%	4 h	70%
6	2x40 µM	20%	MCD, 10%	4 h	75%
7	2x40 µM	20%	MCD, 10%	24 h	89%
8	2x40 µM	20%	HPCD, 10%	24 h	0%
